# Stereotactic body radiotherapy as metastasis-directed therapy in oligometastatic prostate cancer: a systematic review and meta-analysis of randomized controlled trials

**DOI:** 10.1186/s13014-024-02559-7

**Published:** 2024-12-17

**Authors:** Astrid E. Persson, Andreas Hallqvist, Louise Bjørn Larsen, Mette Rasmussen, Jonas Scherman, Per Nilsson, Hanne Tønnesen, Adalsteinn Gunnlaugsson

**Affiliations:** 1 Division of Oncology, Department of Clinical Sciences, Faculty of Medicine, Lund University, Lund, Sweden; 2 Department of Hematology, Oncology, and Radiation Physics, Skåne University Hospital, Lund, Sweden; 3 Department of Oncology, Institute of Clinical Sciences, Sahlgrenska Academy, University of Gothenburg, Gothenburg, Sweden; 4 Department of Oncology, Sahlgrenska University Hospital, Gothenburg, Sweden; 5 Department of Oncology, Herlev Hospital, Copenhagen University Hospitals, Herlev, Denmark; 6 National Institute of Public Health, University of Southern Denmark, Copenhagen, Denmark; 7 Clinical Health Promotion Centre, Department of Health Sciences, Lund University, Lund, Sweden; 8 Clinical Health Promotion Centre, WHO Collaborating Centre, The Parker Institute, Bispebjerg and Frederiksberg Hospital, Copenhagen University, Copenhagen, Frederiksberg, Denmark

**Keywords:** Oligometastatic disease, Prostate cancer, Metastasis-directed therapy, Stereotactic body radiotherapy, Systematic review, Meta-analysis, Randomized controlled trials

## Abstract

**Background:**

The use of stereotactic body radiotherapy (SBRT) to definitively treat oligometastases in prostate cancer has drawn large clinical and research interests within radiation oncology. However, the evidence is considered in its early stages and there is currently no systematic review of randomized controlled trials (RCTs) in this field. We aimed to evaluate the efficacy and safety of SBRT as metastasis-directed therapy (MDT) in oligometastatic prostate cancer (OMPC) compared to no MDT reported in RCTs.

**Methods:**

MEDLINE, Embase, CINAHL Complete, and Cochrane Library were searched on October 28, 2023. Eligible studies were RCTs comparing SBRT as MDT with no MDT in extracranial OMPC, without restrictions on follow-up time, publication status, language, or year. Participant subsets fulfilling the eligibility criteria were included. Critical outcomes were overall survival and grade ≥ 3 toxicity, and additional important outcomes were progression-free survival (PFS), local control, grade 5 toxicity, health-related quality of life, and systemic therapy-free survival. Meta-analyses were planned. Risk of bias was assessed using the Cochrane risk-of-bias tool version 2, and the quality of evidence using the Grading of Recommendations Assessment, Development, and Evaluation.

**Results:**

In total, 1825 unique study reports were identified and seven phase II RCTs with 559 eligible participants were included. Four trials included multiple types of primary cancer. Outcome definitions were heterogeneous except for overall survival and toxicity. For overall survival, only one study reported events in both arms. Meta-analysis of the grade ≥ 3 toxicity results from two trials showed no difference (pooled risk ratio 0.78, 95% confidence interval 0.37–1.65, *p* = 0.52). Four trials reported significantly longer PFS, with a pooled hazard ratio of 0.31 (95% confidence interval 0.21–0.45, *p* < 0.00001). Risk of bias was of some concerns or high. Quality of evidence was low or moderate.

**Conclusions:**

Phase II trials have shown promising improvements in PFS for several OMPC states without excess toxicity. Overall survival comparisons are immature. In future confirmatory phase III trials, adequately large sample sizes, blinding of outcome assessors, and/or increased adherence to assigned intervention could improve the quality of evidence.

*PROSPERO registration number:* CRD42021230131.

**Supplementary Information:**

The online version contains supplementary material available at 10.1186/s13014-024-02559-7.

## Background

Comprehensive treatment of a few metastases, or oligometastases, using metastasis-directed therapy (MDT) has several theoretical benefits. It may prevent or delay metastasis-related complications, postpone the need for systemic therapy or a change of therapy by treating isolated nonresponding metastases [1], interfere with metastatic seeding [2], and be curative if all visible lesions represent the total tumor burden [3].

Stereotactic body radiotherapy (SBRT) is an appealing MDT by being noninvasive and delivered on an outpatient basis [4], and by potentially enhancing the anticancer immune response [5]. SBRT has shown encouraging safety and early efficacy across multiple primary cancer types, with one-year local control rates at approximately 95% [6].

There have been significant efforts in the radiation oncology community during the last years in conducting clinical trials on SBRT as MDT [7], as well as in harmonizing the nomenclature and differentiating clinical scenarios where oligometastatic disease (OMD) is encountered. In 2019, the International Commission on Radiation Units and Measurements (ICRU) published its report [8] on stereotactic radiotherapy (RT). In 2020, the European Society for Radiotherapy and Oncology (ESTRO)–European Organization for Research and Treatment of Cancer (EORTC) consensus recommendation [9] charted OMD states and was shortly followed by the ESTRO–American Society for Radiation Oncology (ASTRO) consensus document [10] specifying prioritized outcomes applicable to SBRT as MDT.

Parallel to these efforts, understanding if SBRT as MDT is beneficial in a particular primary cancer type, and if so, determining its place in the current treatment armamentarium, are essential [11]. Prostate cancer (PCa) is one of the most studied primary cancer types [6, 7]. MDT with SBRT is increasingly offered for oligometastatic prostate cancer (OMPC) in routine clinical practice [12, 13], although the evidence is considered weak so far [14]. In response, ESTRO recently published recommendations [15] on patient selection, imaging, and SBRT delivery for clinical practice and acknowledged the lack of level I evidence.

Several systematic reviews have covered MDT in OMPC, including both retrospective and prospective [16–23] and exclusively prospective [24–27] studies. Among the systematic reviews of prospective studies, some covered multiple types of MDT (i.e., also surgery) [24, 26] and SBRT alone [25, 27].

However, to our knowledge, there is not yet a systematic review on SBRT in OMPC limited to randomized controlled trials (RCTs), which provides the highest level of evidence [28]. Therefore, we conducted a systematic review of RCTs investigating the efficacy and safety of SBRT as MDT in OMPC. The main hypothesis was that this intervention would improve overall survival without increasing grade ≥ 3 toxicity compared to a control group (CG) receiving standard of care or no treatment.

## Methods

The protocol was developed according to the Preferred Reporting Items for Systematic Reviews and Meta-analyses (PRISMA) guidelines [29] and pre-registered in PROSPERO (CRD42021230131) [30]. The following report is written according to the PRISMA 2009 guidelines [31].

### Eligibility criteria

We included RCTs that investigated SBRT as MDT for extracranial OMPC. The participants were adults with OMPC diagnosed through biopsy of the primary tumor or metastasis and positron emission tomography (PET)-computed tomography (CT) or whole body magnetic resonance imaging (MRI) [32], or according to the study authors. Studies that included only patients with regional nodal metastases were ineligible. However, participant subsets fulfilling the eligibility criteria were included, such as those from a study that investigated several primary cancer types.

All metastases, or all uncontrolled metastases as a minimum in oligoprogressive disease, had to be treated with SBRT without other concomitant MDTs in the intervention group (IG). SBRT could be referred to using related terms (e.g., stereotactic ablative RT or radiosurgery), with the reservation of dose per fraction being > 2 Gy according to the ICRU definition [8], and could be one of several investigated treatments.

The CG was receiving standard of care (SOC), placebo, no treatment, or RT with lower intensity than the IG as part of the study design. Lower intensity RT was defined as standard palliative RT or equivalent dose in 2-Gy fractions (EQD2) of up to 50 Gy (α/β = 3 Gy) to any metastasis. Higher doses were allowed at the physicians’ discretion during follow-up.

Each study report had to report at least one of the review outcomes (see Outcomes). There were no restrictions on follow-up time and publication status, language, or year of publication. Original study reports and other publication formats (e.g., editorials, comments, and letters) were eligible. Studies with insufficient data for qualitative or quantitative analysis were excluded.

### Outcomes

The review outcomes were clinical outcomes from the ESTRO–ASTRO consensus document on metastasis-directed RT in OMD [10] and were grouped into critical, additional important, and other relevant outcomes. Critical outcomes were: overall survival, defined as time from randomization to death from any cause, or according to the study authors; and incidence proportion of grade ≥ 3 toxicity, defined as the proportion of participants with at least one event of grade ≥ 3 on the Radiation Therapy Oncology Group/EORTC toxicity scale [33] or Common Terminology Criteria for Adverse Events (CTCAE) version 5.0 [34] after start of SBRT, or according to the study authors, at 5 years or longest follow-up.

Additional important outcomes were progression-free survival (PFS), defined as time from randomization to disease progression according to the Response Evaluation Criteria in Solid Tumors (RECIST) version 1.1 (i.e., ≥ 20% increase in the lesions’ baseline sum diameter with a minimum absolute increase of 5 mm for measurable disease and unequivocal progression or progression warranting a change in therapy for nonmeasurable disease) [35], or according to the study authors; local control, defined as the proportion of participants without locally progressive disease according to RECIST 1.1 or according to the study authors, at 5 years or longest follow-up; incidence proportion of grade 5 toxicity, or lethality ascribed to treatment, after start of SBRT or as defined by the study authors, at 5 years or longest follow-up; health-related quality of life (HRQoL), assessed by the EuroQol Group’s EQ-5D-5L index value [36], 36-Item Short Form Health Survey (SF-36) physical or mental component summary [37], EORTC Quality of Life Questionnaire-Core 30 (QLQ-C30) version 3 summary score [38, 39] with or without the complementary module Quality of Life Questionnaire-Prostate 25 (QLQ-PR25) [40], Functional Assessment of Cancer Therapy-General (FACT-G) version 4 total score [41], Functional Assessment of Cancer Therapy-Prostate (FACT-P) version 4 total score [42], or according to the study authors, at 3 months; HRQoL at 5 years or longest follow-up; and systemic therapy-free survival, defined as time from randomization to initiation of any systemic anticancer therapy or death from any cause, or according to the study authors. Five-year follow-up was used if reported for the outcomes defined at these timepoints; otherwise, the longest follow-up was reported. Timepoints of 1–6 months were eligible for HRQoL at 3 months.

Other relevant outcomes were local PFS, defined as time from randomization to progression of metastases present at baseline according to RECIST 1.1 or according to the study authors; and distant PFS, defined as time from randomization to occurrence of a new metastatic lesion according to RECIST 1.1 or according to the study authors.

### Search strategy

MEDLINE (through PubMed), Embase (through Embase.com), Cumulative Index to Nursing and Allied Health Literature (CINAHL) Complete (through EBSCOhost), and Cochrane Central Register of Controlled Trials (CENTRAL) were searched for study reports. Additionally, the ClinicalTrials.gov and International Clinical Trials Registry Platform (ICTRP) were searched for protocols to identify ongoing studies.

Other sources were also screened for study reports: reference lists of relevant systematic reviews, abstract collections, backward and forward citations searching on included study reports using Web of Science, authors of the included studies, and selected vendor companies. Relevant systematic reviews were identified by applying systematic review filters in the database searches, except for CENTRAL where Cochrane Database of Systematic Reviews was searched instead.

The first searches covered the period from inception until January 24, 2021, and were rerun on October 28, 2023, when also previously identified ongoing studies were checked for completion. The full search strategy is provided in Additional file 1.

### Study selection

The identified study reports were imported into EndNote [43] where duplicates were removed according to the deduplication strategy described in Additional file 2. AEP and AG independently screened unblinded titles and/or abstracts in duplicate in Covidence [44] for potentially eligible study reports. Disagreements were resolved through discussion; otherwise, HT made the final decision. AEP screened other sources.

Study reports were collated at the study level, and a main report was chosen. AH and LBL independently assessed unblinded full texts in duplicate for inclusion and decided the main reason for exclusion based on the order of importance listed in Additional file 3. Disagreements were resolved by discussion; otherwise, AG was consulted and could make the final decision. Study reports and duplicates could be included or excluded until manuscript submission.

The study authors were contacted for clarification of the eligibility criteria, if needed. If no clarification could be obtained, the main report was used in case of conflicting information and if information was missing, the study was excluded. Ongoing and seemingly eligible studies without any reported outcome data were to be described in separate tables.

### Data collection

Data were collected from unblinded study reports using Google Forms [45]. We used full reports, their supplements, and abstracts, as well as protocols when relevant for study definitions, and examined the latest protocol in the case of multiple versions. If results were reported only in figures, data were extracted using Engauge Digitizer [46]. AEP collected data on study characteristics, while AH and LBL collected outcome data using forms piloted on one included study. Discrepancies were resolved by discussion; otherwise, AG was consulted and could make the final decision. The study authors were contacted for missing or additional data.

The critical and important outcomes were assessed for risk of bias using the Cochrane risk-of-bias tool version 2 (RoB 2) [47] that considers five domains: randomization process, deviations from intended interventions, missing outcome data, measurement of the outcome, and selection of reported result, on the study and outcome level. AH and LBL independently performed the unblinded assessments in duplicate. Disagreements were resolved by discussion; otherwise, with AEP, HT, and AG until consensus was reached. The assessments were reported in a traffic light plot according to the Robvis tool [48].

The quality of evidence for the critical and important outcomes was assessed using the Grading of Recommendations Assessment, Development, and Evaluation (GRADE) [49] by applying the same process described above for risk of bias. The assessments were to be reported in the Summary of findings table generated in GRADEpro Guideline Development Tool [50].

### Data items

We extracted data on publication details (e.g., publication year and corresponding author), study details (e.g., design and phase, enrollment regions and country/countries, enrollment period, screening procedures, follow-up period and time, funding, and inclusion and exclusion criteria), diagnostic details (e.g., imaging modalities and OMD criteria), participant details (e.g., age, performance status, baseline prostate-specific antigen [PSA], controlled primary tumor, states of OMD, previous use and type of systemic treatment, previous use and type of MDT, and sites and number of treated metastases), intervention details (e.g., content, timing, methods of delivery, doses, number of and time between treatments, length of intervention, and intervention integrity), and outcomes.

During the review process, we added data on randomization procedures and ratio, follow-up procedures, castration-resistant disease, PSA doubling time, and number of progressing metastases in the CG in case of oligoprogressive disease.

### Data analysis

The hazard ratio (HR) was the effect measure for time-to-event outcomes (overall survival, PFS, systemic therapy-free survival, and local and distant PFS), the risk ratio (RR) for dichotomous outcomes (toxicity and local control), and the mean difference or standardized mean difference for continuous outcomes (HRQoL). Outcome data reported as intention-to-treat were used.

Overall survival and incidence proportion of grade ≥ 3 toxicity at 5 years or longest follow-up were going to be analyzed in meta-analysis using Review Manager 5.4.1 [51] and presented in forest plots. The random-effects model was chosen because of the expected large clinical heterogeneity among studies. If a study reported no events in neither the IG nor the CG, it was excluded from the meta-analysis. A meta-analysis would not be performed if the clinical or statistical heterogeneity were too high.

For overall survival, a pooled HR with a 95% confidence interval (CI) was going to be estimated using the generic inverse-variance method based on the natural logarithm (ln) of the HR and the variance of the ln(HR) [52]. In case these statistics were not reported, they were estimated using direct or indirect methods described by Tierney et al. [53]. For incidence proportion of grade ≥ 3 toxicity at 5 years or longest follow-up, a pooled RR with a 95% CI was going to be estimated using the DerSimonian and Laird inverse-variance method. The pooled effect estimates were to be tested for an overall effect using the Z-test with a significance level of 0.05.

Statistical heterogeneity was to be assessed using the Chi^2^ test with a significance level of 0.10. The proportion of observed variation in effects between studies reflecting real statistical heterogeneity was to be estimated using I^2^ with a 95% CI and quantified using Tau^2^, the variance in true effects between studies, with a 95% CI. I^2^ of ≥ 50% was considered to represent substantial heterogeneity. Prediction intervals were to be calculated in case ≥ 10 studies were included in a meta-analysis [52]. Sensitivity analyses excluding studies with high risk of bias were planned.

Formal assessment of publication bias was planned if ≥ 10 studies were included in the meta-analyses by generating funnel plots and testing for asymmetry using Egger’s test with a significance level of 0.10 [54].

All outcome data were reported narratively and in tables. The additional important outcomes were also to be reported using pooled effect estimates.

## Results

### Study selection

**Fig. 1:**
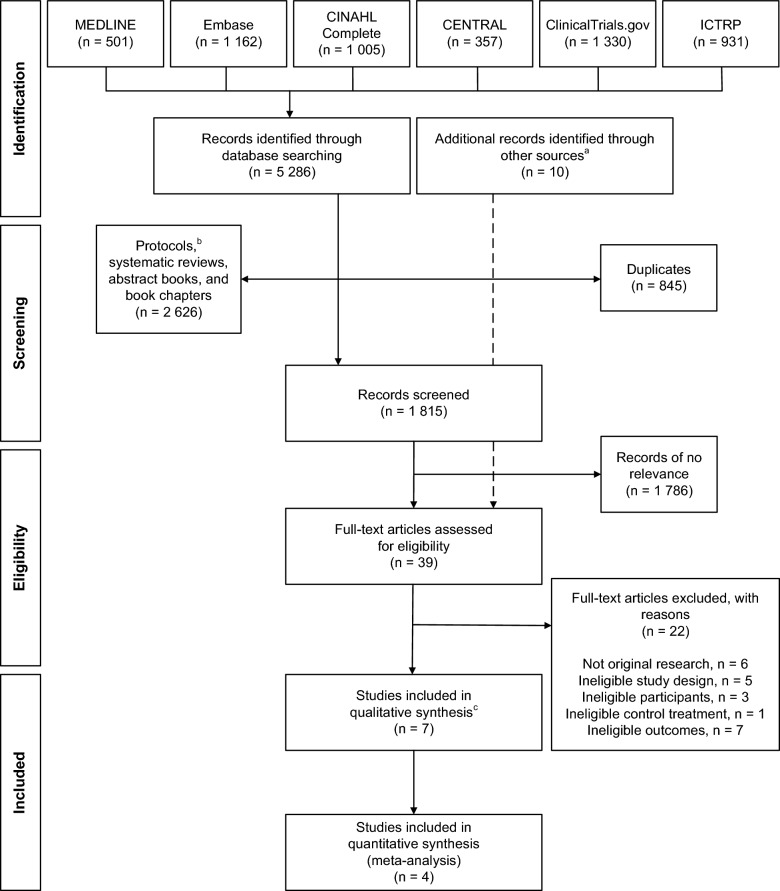
Modified PRISMA 2009 flow diagram. Flow diagram of study reports during study selection. *CENTRAL* Cochrane Central Register of Controlled Trials, *CINAHL* Cumulative Index to Nursing and Allied Health Literature, *ICTRP* International Clinical Trials Registry Platform. ^a^Potentially eligible study reports from reference lists of relevant systematic reviews, abstract collections, backward and forward citations searching on included study reports, authors of included studies, and selected vendor companies ^b^Twelve additional protocols were identified from other sources ^c^Reported in 17 study reports

We identified 5286 records from the database searches. After removing duplicates, protocols, systematic reviews, abstract collections, and book chapters, 1815 titles and/or abstracts were screened. After adding 10 records identified from other sources, 39 full texts were assessed for inclusion. Seven RCTs reported in 17 study reports were included. Three of the reports were identified from other sources: two by personal knowledge of the review authors and one by tracking a later report of a pilot study. The flow diagram of the study selection process is shown in Fig. 1.

Studies excluded during full-text assessment are shown in Table 1. Eighteen relevant ongoing studies were identified and are described in Additional file 4.

**Table 1:** Characteristics of excluded studies after full-text assessment

Author (Year)	Reason for exclusion
Bazyar (2023)^a^ [55]	Ineligible outcomes
Bowden (2019) [56]	Ineligible study design
Broomfield (2014) [57]	Not informing on original research
De Bleser (2020)^b^ [58]	Ineligible outcomes
Dunst (2018) [59]	Not informing on original research
Dunst (2021) [60]	Not informing on original research
Gupta (2019) [61]	Not informing on original research
Hemmatazad (2021) [62]	Not informing on original research
Hermann (2021) [63]	Not informing on original research
Horjeti (2023)^a^ [64]	Ineligible outcomes
Hrinivich (2019)^a^ [65]	Ineligible study design
Kumar (2021)^d^ [66]	Ineligible outcomes
Nguyen (2019)^c^ [67]	Ineligible participants
Qu (2020)^d^ [68]	Ineligible outcomes
Qu (2021)^d^ [69]	Ineligible outcomes
Raymakers (2021)^d^ [70]	Ineligible outcomes
Ryu (2019) [71]	Ineligible participants
Siva (2020)^c^ [72]	Ineligible study design
Sprave (2018)^c^ [73]	Ineligible participants
Sridharan (2016)^e^ [74]	Ineligible study design
van de Ven (2020) [75]	Ineligible study design
Zelefsky (2021) [76]	Ineligible control group

^a^Include data from ORIOLE

^b^Include data from STOMP

^c^Information for eligibility assessment provided by personal correspondence with study authors

^d^Include data from SABR-COMET

^e^Include data from TROG 03.04 RADAR

### Study characteristics

The included trials were STOMP [77–79], SABR-COMET [80–83], ORIOLE [79, 84], ARTO [85–90], EXTEND [91], CORE [92], and STOP [93], which were published between 2017 and 2023. The trials included 559 participants with OMPC fulfilling our eligibility criteria: 200 in the IGs, 179 in the CGs, and an additional 180 participants in both groups in CORE. All were phase II multicenter trials conducted in high-income Western countries. Their characteristics are listed in Table 2 and further details are provided in Additional file 5. After contacting the authors of all included studies, we were able to receive supplementary data from the ARTO team.

**Table 2:** Characteristics of included studies

Study *Author (Year)* Countries	№ participants IG *vs* CG	№ centers	Follow-up time in months; median	Imaging modalities	Age in years; median	Metastases № (Categories)	Controlled primary tumor	Castration-resistant disease	State(s) of OMD^a^	Intervention Total radiation dose (Fractionation)	Control
STOMP [77–79] *Ost (2017, 2020),* *Deek (2022)* Belgium	25 (subset^b^) *vs* 31	6	NI	Choline PET-CT	NI	1–3 (Node, bone, or both)	Yes	No	Metachronous oligorecurrence	SBRT 30 Gy (10 Gy × 3) EQD2_3_ 78 Gy	Surveillance
SABR-COMET [80, 83] *Palma (2019, 2020),* *Olson (2019), Harrow (2022)* Canada, the Netherlands, UK, Australia	14 *vs* 2 (both subsets^c^)	10	NI	CT and bone scan or PET-CT^d^ Spine MRI if vertebral metastases	NI	1–5; ≤ 3 per organ (Node, bone, lung, liver, adrenal, pararenal)	Yes	Mixed^P^	Any^P^	SBRT added to SOC 16–60 Gy (5–20 Gy × 1–12)^P^ EQD2_3_ range 61–227 Gy	SOC
ORIOLE [79, 84] *Phillips (2020),* *Deek (2022)* USA	36 *vs* 18	3	18.8 (range 5.8–35.0)	CT, MRI, and/or bone scan	IG: 68 (range 61–70) CG: 68 (64–76)	1–3 (Node, bone, or both)	NI	No	Metachronous oligorecurrence	SBRT 19.5–48.0 Gy (5–12 Gy × 3–5) EQD2_3_ 37–144 Gy	Observation
ARTO [85–90] *Francolini (2020, 2021, 2022a, 2022b, 2023a, 2023b)* Italy	75 *vs* 82	16	24.9 (IQR 17.1–35.8)	CT and/or bone scan or choline, fluciclovine, or PSMA PET-CT	IG: 74 (IQR 68–79) CG: 74 (68–79)	1–3 (Node, bone, or both)	9% without^e^	Yes	Any with castration-resistance	SBRT 16–40 Gy (6.5–16 Gy × 1–5) EQD2_3_ 61–108 Gy Abiraterone acetate + prednisone, ADT	Abiraterone acetate + prednisone, ADT
EXTEND [91] *Tang (2023)* USA	43 *vs* 44	3	22.0 (range 11.6–39.2)	CT and bone scan or fluciclovine PET-CT	IG: 67 (IQR 63–72) CG: 67 (63–72)	1–5 metastases (Node, bone ± node, other sites ± bone, node)	Yes^f^	Mixed IG: 9% CG: 7%	Any	SBRT (MDT^g^) Recommended, 12–70 Gy (2.3–27 Gy × 1–28)^P^ EQD2_3_ 17–162 Gy Intermittent hormone therapy	Intermittent hormone therapy
CORE [92, 115] *Khoo (2023)* UK, Australia	Total 180 (subsets in both arms^h^)	30	NI	CT and bone scan or choline/PSMA PET-CT or WBMRI^P^	NI	1–3 in 1–2 organs^P^	Yes^P^	Mixed^P^	Metachronous oligorecurrence	SBRT added to SOC Recommended, 24–60 Gy (7.5–18 Gy × 3–8)^P^ EQD2_3_ 53–227 Gy	SOC
STOP [93, 116] *Schellenberg (2023)* Canada	7 *vs* 2 (both subsets^e,i^)	8	NI	NI	NI	1–5 progressing; ≤ 3 per organ^P^	NI	33% hormone-sensitive^e^	Oligoprogression	SBRT to all progressing metastases added to SOC	SOC

Reported in table as described by the study authors and denoted by superscript P if only reported in protocol. Equivalent dose in 2-Gy fractions (EQD2) for α/β = 3 Gy

*ADT* androgen deprivation therapy, *CG* control group, *CT* computed tomography, *EORTC* European Organization for Research and Treatment of Cancer, *ESTRO* European Society for Radiation Oncology, *Gy* Gray, *IG* intervention group, *IQR* interquartile range, *MDT* metastasis-directed therapy, *MRI* magnetic resonance imaging, *NI* no information, *OMD* oligometastatic disease, *PET* positron emission tomography, *PSMA* prostate-specific membrane antigen, *SBRT* stereotactic body radiotherapy, *SOC* standard of care, *vs* versus, *WBMRI* whole body magnetic resonance imaging

^a^According to ESTRO–EORTC consensus recommendation

^b^Six additional participants in the IG were treated with surgery only as decided by the multidisciplinary team

^c^Eighty-three additional participants with other primary cancer types were included in the study

^d^PET-CT was required for solitary pulmonary nodules not pathologically verified, or could be done as decided by the treating oncologists

^e^Information provided by personal correspondence with study authors

^f^Participants with untreated primary tumors received prostate radiotherapy on trial

^g^Some participants may have received conventional fractionation only, e.g., due to regional nodal disease

^h^Sixty-five additional participants with other primary cancer types were included in the study

^i^Eighty-one additional participants with other primary cancer types were included in the study

STOMP, ORIOLE, and ARTO included only participants with PCa, whereas SABR-COMET, EXTEND, CORE, and STOP included several primary cancer types. In the included EXTEND study report, the PCa basket receiving intermittent hormone therapy was reported separately. In STOMP, ORIOLE, and CORE, only patients with metachronous oligorecurrence were included, and in STOP, patients with oligoprogression.

OMD was defined as 1–5 (progressing) metastases detected using CT and/or bone scan, MRI, or PET-CT with choline, fluciclovine, or PSMA tracers, with some variations in restrictions on metastatic sites and the number of metastases per organ. It was uncertain if the participants had brain metastases in SABR-COMET, EXTEND, and STOP, but it was considered unlikely per personal correspondence with the study author for SABR-COMET and by the review authors for EXTEND and STOP. Only participants with castration-resistant disease were studied in ARTO and were eligible for enrollment in SABR-COMET, EXTEND, CORE, and STOP.

STOMP investigated either SBRT or surgery as MDT, while the remaining trials investigated SBRT. There was some uncertainty as to whether some participants in EXTEND had received only conventionally fractionated RT, e.g., due to regional nodal disease; however, the proportion of participants with regional nodal disease (7%) was balanced between the arms. The fractionation schedules that were or could be used varied between total doses of 12–70 Gy, with fraction doses of 2.3–27 Gy in 1–28 fractions (EQD2_3_ range 17–227 Gy).

SBRT was delivered without systemic therapy in STOMP and ORIOLE, while the other trials either investigated or allowed systemic therapy in both arms: abiraterone acetate, prednisone, and androgen deprivation therapy (ADT) was added in ARTO; intermittent hormone therapy (ADT with or without a second-generation androgen receptor inhibitor for ≥ 6 months with planned break 4–8 months after enrollment) in EXTEND; and SOC in SABR-COMET, CORE, and STOP.

### Results of individual studies

The outcomes fulfilling the review criteria and the corresponding data are listed in Table 3. In one study [84], crossover to the intervention arm was allowed at progression or 6 months, that is, the timepoint of the trial’s primary outcome. Therefore, we only included follow-up until 6 months in the analysis of this trial.

**Table 3:** Outcome data presented as effect estimates (95% confidence intervals), medians (95% confidence intervals), № (%)

Systematic review outcome	STOMP [77, 79]	SABR-COMET [80–83]	ORIOLE [79, 84]	ARTO [85, 90]	EXTEND [91]	CORE [92, 115, 117]	STOP [93, 116]
*Overall survival*							
Outcome name	Overall survival	Overall survival	Overall survival	Overall survival	Overall survival	NP	Overall survival
Definition	Time to death^P^	Time to death	Time to death^P^	Time to death	Time to death^P^		Time to death^P^
Follow-up	–	–	6 months until crossover	24.9 months (IQR 17.1–35.8)	22.0 months (range 11.6–39.2)		–
IG			Median not reached (i.e., < 50% with event)	Median not reached	Median not reached		
CG			Median not reached	Median not reached	Median not reached		
Results		Model does not converge	No events in either group^a^ [79]	HR 0.65 (0.28–1.49) *p* = 0.302	“Overall survival data were immature, […] similar between arms,” log-rank *p* = 0.21^a^ [91]		
*Incidence proportion of grade* ≥ *3 toxicity at 5 years or longest follow-up*							
Outcome name	Toxicity grade ≥ 3	Toxicity grade ≥ 3	Adverse effects grade ≥ 3	Adverse events grade ≥ 3	Adverse events grade ≥ 3	NP	Toxicity grade ≥ 3 related to RT^P^
Definition	CTCAE v4.0	CTCAE v4	CTCAE v4.0	CTCAE v4.03	CTCAE v4.0		CTCAE v4 for each treated organ^P^
Follow-up	–	–	6 months until crossover	24.9 months (IQR 17.1–35.8)	22.0 months (range 11.6–39.2)		–
IG	0/25 (0%)		0/36 (0%)	8/75 (11%)	3/43 (7%)		
CG	0/31 (0%)		0/18 (0%)	13/82 (16%)	2/44 (5%)		0/2 (0%)
Results	NA		NA	RR 0.67 (0.30–1.53) *p* = 0.345^b^	RR 1.53 (0.27–8.74) *p* = 0.629^b^		NA
*PFS*							
Outcome name	PFS	PFS	PFS	PFS	PFS	PFS	PFS
Definition	Time to PSA/clinical/ radiographic progression,^c^ start of ADT, death, or study withdrawal	Time to progression according to RECIST 1.0^P^ or death	Time to PSA/clinical/ radiographic progression,^c^ start of ADT, death, or study withdrawal	Time to PSA/radiographic progression,^d^ start of following treatment, or death	Time to PSA/clinical/radiographic progression^e^ or death	Time to radiographic ± PSA/clinical progression^f^ or death^P^	Time to progression or death^P^
Follow-up	–	–	6 months until crossover	24.9 months (IQR 17.1–35.8)	22.0 months (range 11.6–39.2)	–	–
IG			Median not reached	Median not reached	Median not reached		
CG			Median not reached	17 months	15.8 months (13.6–21.2)		
Results		HR 0.09 (0.01–0.65) *p* = 0.032^g^	HR 0.21 (0.07–0.61) *p* = 0.004^a^ [84]	HR 0.35 (0.21–0.57) *p* < 0.001	HR 0.25 (0.12–0.55) *p* < 0.001		
			HR 0.35 (0.14–0.88) *p* = 0.025^a^ [79]				
*Local control at 5 years or longest follow-up*							
Outcome name	Symtomatic or local progression	Lesional control rate	NP	NP	NP	NP	Lesional control
Definition	See footnote^h^	RECIST 1.0^P^					–
Follow-up	–	–					
IG	0/– occurrences						
CG	9/– occurrences						
Results	NA						
*Incidence proportion of grade 5 toxicity at 5 years or longest follow-up*							
Outcome name	Toxicity grade 5	Toxicity grade 5	Adverse effects grade 5	Adverse events grade 5	Adverse events grade 5	NP	Toxicity grade 5 related to RT^P^
Definition	CTCAE v4.0	CTCAE v4	CTCAE v4.0	CTCAE v4.03	CTCAE v4.0		CTCAE v4 for each treated organ^P^
Follow-up	-	-	6 months until crossover	24.9 months (IQR 17.1–35.8)	22.0 months (range 11.6–39.2)		-
IG	0/25 (0%)	–	0/36 (0%)	0/75 (0%)	0/43 (0%)		0/7 (0%)
CG	0/31 (0%)		0/18 (0%)	0/82 (0%)	0/44 (0%)		0/2 (0%)
Results	NA		NA	NA	NA		NA
*HRQoL at 3 months*							
Outcome name	Quality of life	Quality of life	Quality of life	Quality of life	Quality of life	NP	NP
Definition	EORTC QLQ-C30, QLQ-PR25	FACT-G	BPI-SF	EORTC QLQ-C30	CES-D, MDASI, SF-12		
Follow-up	3 months	6 months	3, 6 months^P^	3 months	3 months^P^		
IG	–	–	–	Global Health Status: Mean 63.3 (13 participants)^i^	–		
CG	Global Health Status: Mean 78 (SD 18)^a^ [77]			Mean 63.3 (16 participants)^i^			
Results	–		No difference between groups, data not reported	–			
*HRQoL at 5 years or longest follow-up*							
Outcome name	Quality of life	Quality of life	NP	Quality of life	Quality of life	NP	Quality of life
Definition	EORTC QLQ-C30, QLQ-PR25	FACT-G		EORTC QLQ-C30, BPI-SF	CES-D, MDASI, SF-12		FACT-G^P^
Follow-up	1 year	6 years		1 year^P^	22.0 months (range 11.6–39.2)		5 years^P^
IG	–	–		–	–		–
CG	Global Health Status: Mean 75 (SD 20)						
Results	–				No difference between groups according to linear mixed-effects modelling		
*Systemic therapy-free survival*							
Outcome name	ADT-free survival	NP	NP	NA^j^	NA^j^	NP	NP
Definition	Time to start of palliative ADT or death						
Follow-up	–						
IG							
CG	13 months (80% CI 12–17)						
Results	–						
*Local PFS*							
Outcome name	NP	NP	NP	NP	NP	NP	NP
Definition							
Follow-up							
IG							
CG							
Results							
*Distant PFS*							
Outcome name	Radiographic PFS	Time to development of new metastases	Radiographic PFS	NP	Time to new lesion failure	NP	NP
Definition	Time to new nodal, bone, or visceral metastasis or death		Time to new nodal, bone, or visceral metastasis or death		Time to new lesion outside of the initial lesions present^P^		
Follow-up	–	–	6 months until crossover		2 years		
IG			–		Median not reached^a^ [91]		
CG							
Results					HR 0.33 (0.11–1.0) *p* = 0.04, favoring the IG		

Reported as described by the study authors and denoted by superscript P if only reported in protocol. Dash: No data/No information

*ADT* androgen deprivation therapy, *BPI-SF* Brief Pain Inventory (Short Form), *CES-D* Center for Epidemiological Studies-Depression, *CG* control group, *CI* confidence interval, *CT* computed tomography, *CTCAE* Common Terminology Criteria for Adverse Events, *EORTC* European Organization for Research and Treatment of Cancer, *FACT-G* Functional Assessment of Cancer Therapy-General, *HR* hazard ratio, *HRQoL* health-related quality of life, *IG* intervention group, *IQR* interquartile range, *MDASI* MD Anderson Symptom Inventory, *MRI* magnetic resonance imaging, *PFS* progression-free survival, *NA* not applicable, *NP* not published, *PSA* prostate-specific antigen, *QLQ-C30* Quality of Life Questionnaire-Core 30, *QLQ-PR25* Quality of Life Questionnaire-Prostate 25, *RECIST* Response Evaluation Criteria in Solid Tumors, *RR* risk ratio, *RT* radiotherapy, *RTOG* Radiation Therapy Oncology Group, *SD* standard deviation, *SF-12* 12-Item Short Form Health Survey, *v* version

^a^Extracted from figure

^b^Estimated from the groups’ results

^c^PSA progression: ≥ 25% and ≥ 2 µg/L above nadir confirmed after ≥ 4 weeks.^P^ Clinical: symptomatic progression. Radiographic: ≥ 20% in soft tissue lesions’ sum diameter per RECIST 1.1 (applied to all lesions when a lesion did not meet criteria) on CT; ≥ 1 new lesion(s)^P^ on bone scan; on MRI; or any evidence by size

^d^PSA progression: rises ≥ 1 week apart and ≥ 2 µg/L. Radiographic: 20% in sum of target lesions’ longest diameters; new soft tissue/visceral lesions; and/or unequivocal progression on CT/MRI^P^

^e^PSA progression: ≥ 25% and ≥ 2 µg/L above nadir. Clinical: treatment change needed.^P^ Radiographic: RECIST 1.1^P^

^f^Radiographic progression: RECIST 1.1. Bone scan and PSA were considered. When progression could not be measured, clinical evidence was used^P^

^g^Estimated from HR and CI

^h^Progression of soft tissue lesions: ≥ 20% and ≥ 5 mm in longest diameter. Bone: ≥ 25% on CT [118]

^i^Data provided by personal correspondence with study authors

^j^Trial treatment included systemic therapy

Outcomes were measured from either date of informed consent, enrollment, randomization, or start of (systemic) treatment as reported in the study reports or protocols. Overall survival and toxicity were homogenously defined as time to death and by applying CTCAE version 4, respectively. The studies used different clinical, biochemical, and/or radiographic definitions of PFS and local control and HRQoL instruments. One study [77, 78] reported systemic therapy-free survival as time to start of ADT. No study has compared the local PFS. Four studies [79, 82, 91] reported distant PFS as time to new metastasis.

Data were published exclusively as figures for overall survival and PFS until 6 months in ORIOLE [79, 84] and for HRQoL at 3 months in STOMP [77], and were carefully estimated from the respective figures. ORIOLE also reported distant PFS exclusively as a figure [79]; however, we refrained from extracting the data owing to uncertainties in estimation around the 6-month mark.

EXTEND [91] reported overall survival exclusively as a figure with a log-rank test. The study authors described that “Overall survival data were immature, […] similar between arms,” with no events in the IG and two events in the CG, with a log-rank *p* of 0.21.

### Synthesis of results

**Fig. 2:**
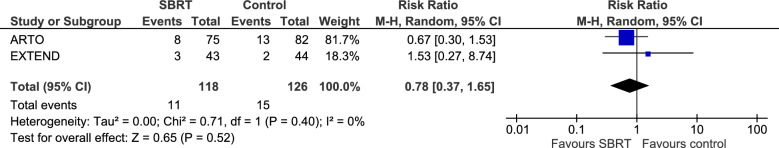
Meta-analysis for incidence proportion of grade ≥ 3 toxicity at 5 years or longest follow-up. Pooled results for critical outcome incidence proportion of grade ≥ 3 toxicity at 5 years or longest follow-up. *CI* confidence interval *df* degrees of freedom, *IV* inverse-variance, *M-H* Mantel–Haenszel method, *SBRT* stereotactic body radiotherapy

A meta-analysis for overall survival was not possible because only one out of three trials reported events in both arms. The trial [90] showed no significant differences between the arms (HR 0.65, 95% CI 0.28–1.49, *p* = 0.302). The pooled results for incidence proportion of grade ≥ 3 toxicity from two trials [90, 91] with events in both arms showed no difference between groups (pooled RR 0.78, 95% CI 0.37–1.65, *Z* = 0.65, *p* = 0.52, Fig. 2). Statistical heterogeneity was not detected.

**Fig. 3:**
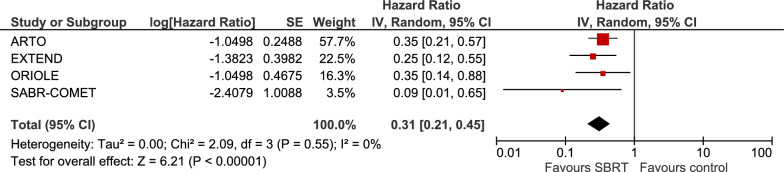
Meta-analysis for progression-free survival**.** Pooled results for additional important outcome PFS. The SABR-COMET trial results were for the prostate cancer subset. *CI* confidence interval, *df* degrees of freedom, *IV* inverse-variance, *PFS* progression-free survival, *SBRT* stereotactic body radiotherapy

The pooled results from four trials [79, 83, 90, 91] showed significantly longer PFS after SBRT, with an HR of 0.31 (95% CI 0.21–0.45, *Z* = 6.21, *p* < 0.00001, Fig. 3). There was no evident statistical heterogeneity. A sensitivity analysis excluding trials with a high risk of bias was not performed because it was not detected in the trials for this outcome (see below).

The critical and additional important outcomes are summarized in Table 4.

**Table 4:** Summary of findings

Stereotactic body radiotherapy as metastasis-directed therapy *vs* no metastasis-directed therapy in oligometastatic prostate cancer						
Patient or population: patients with OMPC Setting: NA and Europe Intervention: SBRT as MDT Comparison: no MDT						
Outcomes	Anticipated absolute effects* (95% CI)		Relative effect (95% CI)	№ of participants (studies)	Certainty of the evidence (GRADE)	Comments
	Risk with no MDT	Risk with SBRT as MDT				
Overall survival follow-up: median 24.9 months	18 per 100^a^ [90]	12 per 100 (5 to 26)	HR 0.65 (0.28 to 1.49) [Death]	157 (1 RCT)	⊕ ⊕ ⊕ ⊝ Moderate^b^	2 additional studies had no events and 2 events in the CG only, respectively
Incidence proportion of grade ≥ 3 toxicity at 5 years or longest follow-up assessed with: CTCAE v4 follow-up: range 6 to 39.2 months	12 per 100	9 per 100 (4 to 20)	RR 0.78 (0.37 to 1.65)	244 (2 RCTs)	⊕ ⊕ ⊝ ⊝ Low^c,d^	2 additional studies had no events for in total 61 participants in the IGs and 49 in the CGs
PFS follow-up: range 5.8 to 39.2 months	59 per 100^e^ [79, 83, 90, 91]	24 per 100 (17 to 33)	HR 0.31 (0.21 to 0.45) [Progression]	314 (4 RCTs)	⊕ ⊕ ⊝ ⊝ Low^c,d^	
Local control at 5 years or longest follow-up	Not reported					^f^
Incidence proportion of grade 5 toxicity at 5 years or longest follow-up assessed with: CTCAE v4 follow-up: range 6 to 39.2 months	0 per 100	0 per 100	not estimable	363 (5 RCTs)	⊕ ⊕ ⊝ ⊝ Low^b,c^	5 studies had no events
HRQoL at 3 months^g^	2 studies reported no differences without further data and mean values only, respectively			141 (2 RCTs)	−	Quality of evidence assessment not performed due to absence of effect estimate
HRQoL at 5 years or longest follow-up	1 study reported no differences according to linear mixed-effects modelling			87 (1 RCT)	–	See above
Systemic therapy-free survival	Not reported					

*The risk in the intervention group (and its 95% CI) is based on the assumed risk in the comparison group and the relative effect of the intervention (and its 95% CI)

*CI* confidence interval, *CTCAE v4* Common Terminology Criteria for Adverse Events version 4, *GRADE* Grading of Recommendations Assessment, Development, and Evaluation, *HR* hazard ratio, *HRQoL* health-related quality of life, *MDT* metastasis-directed therapy, *OMPC* oligometastatic prostate cancer, *PFS* progression-free survival, *RCT* randomized controlled trial, *SBRT* stereotactic body radiotherapy

GRADE Working Group grades of evidence

High certainty: we are very confident that the true effect lies close to that of the estimate of the effect

Moderate certainty: we are moderately confident in the effect estimate: the true effect is likely to be close to the estimate of the effect, but there is a possibility that it is substantially different

Low certainty: our confidence in the effect estimate is limited: the true effect may be substantially different from the estimate of the effect

Very low certainty: we have very little confidence in the effect estimate: the true effect is likely to be substantially different from the estimate of effect

^a^Estimated from number of events in the CG

^b^Downgraded by 1 level for imprecision. Effect estimate based on a single small study

^c^Downgraded by 1 level for risk of bias. Assessors were aware of received intervention and could have influenced assessment

^d^Downgraded by 1 level for imprecision. Few included participants and events; optimal information size criterion was not met

^e^Estimated from number of events in the CGs in the studies included in the meta-analysis. Number of events was estimated from the Kaplan–Meier curve for one study. Another study had no information on number of events and was assumed to have occurred in 1 out of the 2 participants in the CG

^f^Two studies reported occurrences of progression with likely metastasis as unit of analysis instead of participants

^g^Timepoints 1–6 months allowed

### Risk of bias and quality of evidence

**Fig. 4:**
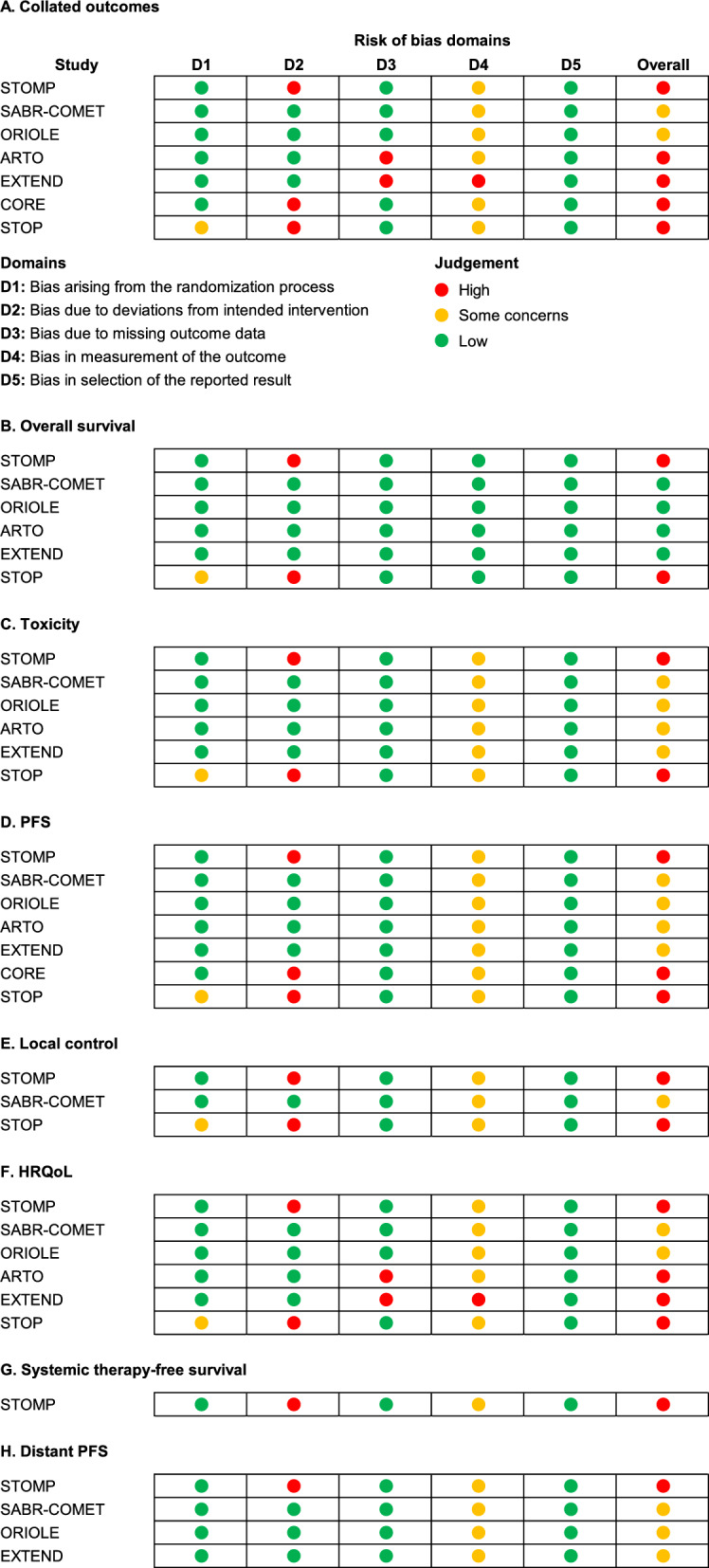
Risk-of-bias assessment for collated outcomes and per reported outcome**.** According to the Cochrane risk-of-bias tool version 2, for **A** Collated outcomes, **B** Overall survival, **C** Toxicity, **D** PFS, **E** Local control, **F** HRQoL, **G** Systemic therapy-free survival, and **H** Distant PFS. High risk of bias was considered due to the following: bias due to deviations from intended intervention—In STOMP and STOP, a part of the CG received the intervention treatment. In CORE, completion of SOC differed between the IG and CG; bias due to missing outcome data—In ARTO and EXTEND, HRQoL assessments were only available for a part of the participants, at 3 months in ARTO and at baseline in EXTEND; and bias due to measurement of the outcome—In EXTEND, HRQoL assessments were optional for participants and willingness to respond may have differed between the arms. *CG* control group, *D* domain, *HRQoL* health-related quality of life, *IG* intervention group, *PFS* progression-free survival, *SOC* standard of care

The risk-of-bias assessment on the study, domain, and outcome levels are shown in Fig. 4, with further comments in Additional file 5.

Overall, the risk of bias arising from the randomization process was low. One trial had some concerns due to no information on allocation concealment; however, only an abstract and trial registry entries were available at that time. Deviations from the intended interventions that could have affected outcomes were observed in three trials. Missing outcome data were considered a risk of bias in two trials, but only for HRQoL outcomes, owing to available data for only some of the trial participants. Bias in measurements of the outcomes was overall of some concerns, except for overall survival, because knowledge of the received intervention could have influenced assessment. However, this was considered unlikely except for HRQoL in one trial where the assessments were optional for the participants and the willingness to respond could have differed between arms.

The quality of evidence assessment is shown in Table 4. The effect estimate for overall survival was considered to have moderate certainty after downgrading by one level for imprecision due to being based on a single study. Toxicity and PFS were considered to have a low certainty and were downgraded by one level each for risk of bias and imprecision. Risk of bias was considered because knowledge of the received intervention could have influenced assessments, and imprecision due to few included participants and events per the GRADE optimal information size criterion [94].

## Discussion

### Summary of evidence

This is, to our knowledge, the first systematic review approaching studies with high-quality designs using randomization and focusing solely on SBRT as MDT in OMPC. We identified seven randomized phase II trials, of which four investigated PCa among several primary cancer types and one investigated SBRT as one of two MDTs (together with surgery).

The results from four studies, one with reported PCa participant subset data, show promising improvements in PFS in several OMD states with a pooled HR of 0.31 (95% CI 0.21–0.45). No excess toxicities or treatment-related deaths were observed. Overall survival comparisons were immature, as only one of the three trials reported events in both arms.

Our findings are in line with those of previous systematic reviews of prospective trials on OMPC that also found favorable disease control and low toxicity [24–27]. All of these systematic reviews included STOMP and ORIOLE, being two early RCTs in this field.

### Strengths and limitations

To summarize the current evidence, we applied generous eligibility criteria allowing participant subsets and various definitions of OMD and SBRT, and performed a comprehensive search of bibliographic databases and other sources. By including eligible participant subsets and updating our searches in the end of 2023, we identified five additional trials with data on OMPC that were not included in previous systematic reviews (i.e., SABR-COMET, ARTO, EXTEND, CORE, and STOP). Reflecting the rapid development in this field, four of these trials were published in the preceding year.

An important outcome of the review is the presentation of the backdrop against which the current evidence in OMPC is generated. The included studies addressed varying patient groups, treatment approaches (e.g., fractionation schedules and co-administered systemic therapies), and control treatments, which limit the conclusions that can be drawn from pooled effect estimates. The use of ADT and other systemic therapies varied between the trials, which need to be considered when interpreting the results. As more RCTs are published, future systematic reviews may be able to give a more granular picture of the treatment’s efficacy by restricting to individual OMD states and by sensitivity to hormonal therapy, a minimally required EQD2, or certain SOC comparators.

Our results are also limited by aspects encountered in emerging fields, that is, evidence being so far generated in small exploratory phase II trials and heterogeneous outcome definitions. Combining studies in a meta-analysis is a strategy to overcome small sample sizes by increasing the statistical power [52]. However, small RCTs, even when pooled, present limitations. When small studies show positive results, they are more likely to exaggerate differences compared with larger trials owing to statistical imprecision [95]. Furthermore, balancing of prognostic factors between the trial arms from randomization can only be assumed if the sample sizes are sufficiently large. It may not be possible to discern if impressive results are due to a prognostic imbalance or a real treatment effect. Therefore, a pooled estimate would be equally uncertain [94, 96]. This was reflected in our quality of evidence assessment by downgrading for imprecision as the GRADE optimal information size criterion calls for meta-analyses of approximately 250 or more events [94].

The outcome definitions were heterogeneous except for overall survival and toxicity. The definitions of PFS included different event criteria and assessment approaches (e.g., different use of PSA, clinical, and radiographic criteria, imaging modalities, and instructions for repeated imaging), which limit comparability across trials. Our pooled effect estimate from the meta-analysis may have differed had one approach been applied in all studies.

Furthermore, our quality of evidence assessment identified two additional challenges in RCTs: blinding of outcome assessors and adherence to assigned intervention. Blinding in trials of radiotherapeutic interventions is inherently difficult and could be considered for radiologists and data analysts. Issues of intervention adherence and crossover have become central as MDT is increasingly being offered in clinical practice. In two of the included studies [77, 93], parts of the CGs eventually received SBRT, and in one study [84], crossover was allowed after the primary endpoint at 6 months, precluding interpretation of most long-term outcomes in this review. Furthermore, one study [92] reported significantly lower completion of SOC in the IG, which could have underestimated the benefit of SBRT.

Crossover to a yet unestablished treatment should be interpreted with caution because it may compete with other treatments. This could prolong systemic therapy-free survival, favoring the CG, or delay effective treatment, favoring the IG, and therefore cloud the comparison in the intention-to-treat population [97]. While the identified toxicity in the literature was low, there are known rare, severe toxicities that may be life-threatening. It is important to introduce these risks only after confirmatory trials have laid the foundation for risk–benefit assessments in comparison with other established palliative treatments. Currently, there is no evidence that MDT is curative for metastatic PCa, thereby necessitating a cautious approach.

### Future research

We identified eight registered and ongoing confirmatory phase III trials that include patients with OMPC (ClinicalTrials.gov registration numbers NCT03143322 [98], NCT03721341 [99], NCT03862911 [100], NCT04115007 [101], NCT04983095 [102], NCT05209243 [103], NCT05717166 [104], and NCT06320067 [105]). One trial including multiple primary cancer types (SABR-COMET-10 [99]) has finalized recruitment, whereas all trials investigating only OMPC are currently recruiting. Of them, the largest is the multi-arm STAMPEDE2 platform [105] with a recruitment target of almost 2500 participants for the comparison of SBRT as MDT added to SOC *vs* SOC.

SBRT as MDT has several important theoretical benefits (e.g., delaying the start of or a change in systemic therapy and enhancing of the anticancer immune response) that need confirmation through an overall survival impact and cannot be fully captured by other outcomes. Such improvements, together with disease control, could benefit patients by minimizing adverse effects, prolonging the usefulness of systemic therapy, and consequently improving HRQoL and optimizing the use of healthcare resources.

Furthermore, the comparator or concomitant treatments become increasingly important in phase III trials for assessing benefits compared with other established treatments. There are several efficacious systemic therapy options for metastatic PCa at different stages of the disease (e.g., ADT, second-generation androgen receptor inhibitors, chemotherapy, poly-ADP ribose polymerase [PARP] inhibitors, and lutetium-177-PSMA-617 [106, 107]), some of which have only been introduced in the past few years, and is also a field of intense research [108]. Similarly, the participants’ previous treatment histories will become equally important to understand the clinical setting in which MDT is investigated. The differences in outcomes between oligometastatic hormone-sensitive and castration-resistant PCa was illustrated in a prospective phase II trial recruiting before the establishment of second-generation androgen receptor inhibitors [109]. After SBRT as MDT and at least two years of ADT, the three-year metastasis progressions-free survival was 67% (95% CI 53–77) in the cohort with hormone-sensitive disease and 26% (95% CI 7–51) in the cohort with castration-resistant disease.

One way to address outcome heterogeneity is core outcome sets (COS), which are standardized sets of outcomes to be minimally measured and reported in trials for a certain health condition [110]. In the database of Core Outcome Measures in Effectiveness Trials (COMET) [111], an initiative promoting and facilitating the use of COS, three sets for research in advanced PCa are available: one with patient-reported symptoms [112], one for metastatic disease in general [113] (published after the start of this review), and one for castration-resistant disease [114]. However, they are not specific to OMD or MDT.

There is a general agreement on important outcome domains, and the ESTRO–ASTRO consensus document [10] specifying outcomes for RT as MDT (used for outcome selection in this review) is a landmark step towards an OMD- and MDT-specific COS. In the future, harmonizing outcome definitions, measurements, and timing could clarify the interpretation of the results and improve trial comparability. Some of these measurements could include qualitative evaluations such as pain and toxicity and would benefit from involving patients, relatives, and healthcare providers during the development process.

Fractionation schedules and EQD2_3_ varied significantly within and between the included studies, introducing another aspect of heterogeneity. Our review highlights the need for careful consideration of the methods for SBRT planning and delivery, including reporting of dose prescription along with relevant dose-volume metrics for targets and organs at risk [8], in future assessments of ongoing research to ensure reproducibility in clinical practice.

## Conclusions

Phase II trials have shown promising improvements in PFS for several OMD states in PCa without excess toxicity. Comparisons for overall survival are currently immature. Outcome definitions were heterogeneous, and harmonization of definitions, measurements, and timing will be essential to the interpretation of results and comparability across trials. The use of outcome measurements as defined in consensus documents is recommended. In future confirmatory phase III trials, adequately large sample sizes, blinding of outcome assessors, and/or adherence to assigned intervention could improve the quality of evidence.

The PRISMA 2009 Checklist is found in Additional file 6.

## Supplementary Information

Additional file 1.

Additional file 2.

Additional file 3.

Additional file 4.

Additional file 5.

Additional file 6.

## Data Availability

Datasets analyzed in this study will be available upon reasonable request to the corresponding author.

## Abbreviations

ADT: Androgen deprivation therapy
ASTRO: American Society for Radiation Oncology
CENTRAL: Cochrane Central Register of Controlled Trials
CG: Control group
CI: Confidence interval
CINAHL: Cumulative Index to Nursing and Allied Health Literature
COMET: Core Outcome Measures in Effectiveness Trials
COS: Core outcome set
CT: Computed tomography
CTCAE: Common Terminology Criteria for Adverse Events
EORTC: European Organization for Research and Treatment of Cancer
EQD2: Equivalent dose in 2-Gy fractions
ESTRO: European Society for Radiotherapy and Oncology
FACT-G: Functional Assessment of Cancer Therapy-General
FACT-P: Functional Assessment of Cancer Therapy-Prostate
GRADE: Grading of Recommendations Assessment, Development, and Evaluation
HR: Hazard ratio
HRQoL: Health-related quality of life
ICRU: International Commission on Radiation Units and Measurements
ICTRP: International Clinical Trials Registry Platform
IG: Intervention group
ln: Natural logarithm
MDT: Metastasis-directed therapy
MRI: Magnetic resonance imaging
OMD: Oligometastatic disease
OMPC: Oligometastatic prostate cancer
PARP: Poly-ADP ribose polymerase
PCa: Prostate cancer
PET: Positron emission tomography
PFS: Progression-free survival
PRISMA: Preferred Reporting Items for Systematic Reviews and Meta-analyses
PSA: Prostate-specific antigen
QLQ-C30: Quality of Life Questionnaire-Core 30
QLQ-PR25: Quality of Life Questionnaire-Prostate 25
RCT: Randomized controlled trial
RECIST: Response Evaluation Criteria in Solid Tumors
RoB 2: Cochrane risk-of-bias tool version 2
RR: Risk ratio
RT: Radiotherapy
SBRT: Stereotactic body radiotherapy
SF-36: 36-Item Short Form Health Survey
SOC: Standard of care
