# Supplementary material for: Stereotactic body radiotherapy as metastasis-directed therapy in oligometastatic prostate cancer: a systematic review and meta-analysis of randomized controlled trials
Source: Radiat Oncol. 2024 Dec 17;19:173. doi: 10.1186/s13014-024-02559-7 (PMC11654405; doi:10.1186/s13014-024-02559-7)
Supplement: Supplementary file 1 — Additional file 1. [file 13014_2024_2559_MOESM1_ESM.doc]

**Additional file 1.** Search strategies

**MEDLINE (through PubMed)**

***Randomized trials***

Search Query

#1 prostat* OR prostate [mh]

#2 neoplasm* OR neoplasms [mh]

#3 tumor* OR tumour*

#4 malign*

#5 cancer*

#6 #2 OR #3 OR #4 OR #5

#7 relaps* OR recur* OR recurrence [mh]

#8 metasta*

#9 #7 OR #8

#10 oligo*

#11 (#1 OR #6) AND #9 AND #10

#12 radiosurg* OR radiosurgery [mh] OR srs

#13 cyberkni* OR "cyber kni*"

#14 ((stereotactic* OR stereotaxic*) AND (radiotherap* OR radiation OR radiotherapy [mh])) OR stereotaxic techniques [mh] OR srt OR sbrt

#15 (ablative* AND (radiotherap* OR radiation OR radiotherapy [mh])) OR radioablati* OR sabr

#16 #12 OR #13 OR #14 OR #15

#17 surviv* OR survival [mh] OR mortalit* OR mortality [mh] OR mortality [sh] OR death* OR death [mh] OR os

#18 toxic* OR toxic actions [mh] OR "side effect*" OR "adverse effect*" OR "adverse event*" OR "adverse outcome*" OR "adverse reaction*" OR adverse effects [sh] OR complication* OR complications [sh]

#19 progress* OR disease progression [mh] OR progression-free survival [mh] OR pfs OR lpfs OR dpfs

#20 control* OR "stable disease*" OR response* OR regress* OR remission* OR remission induction [mh]

#21 "quality of life" OR quality of life [mh] OR qol OR hrqol

#22 "systemic therapy-free survival" OR "androgen deprivation-free survival" OR "androgen deprivation therapy-free survival" OR "ad-free survival" OR "adt-free survival" OR stfs OR adfs OR adtfs

#23 #17 OR #18 OR #19 OR #20 OR #21 OR #22

#24 #11 AND #16 AND #23

#25 randomized controlled trial [pt]

#26 controlled clinical trial [pt]

#27 randomized [tiab]

#28 placebo [tiab]

#29 drug therapy [sh]

#30 randomly [tiab]

#31 trial [tiab]

#32 groups [tiab]

#33 #25 OR #26 OR #27 OR #28 OR #29 OR #30 OR #31 OR #32

#34 animals [mh] NOT humans [mh]

#35 #33 NOT #34

#36 #24 AND #35

#37 systematic [sb]

#38 #24 AND #37

***Systematic reviews***

Search Query

#1 prostat* OR prostate [mh]

#2 neoplasm* OR neoplasms [mh]

#3 tumor* OR tumour*

#4 malign*

#5 cancer*

#6 #2 OR #3 OR #4 OR #5

#7 relaps* OR recur* OR recurrence [mh]

#8 metasta*

#9 #7 OR #8

#10 oligo*

#11 (#1 OR #6) AND #9 AND #10

#12 radiosurg* OR radiosurgery [mh] OR srs

#13 cyberkni* OR "cyber kni*"

#14 ((stereotactic* OR stereotaxic*) AND (radiotherap* OR radiation OR radiotherapy [mh])) OR stereotaxic techniques [mh] OR srt OR sbrt

#15 (ablative* AND (radiotherap* OR radiation OR radiotherapy [mh])) OR radioablati* OR sabr

#16 #12 OR #13 OR #14 OR #15

#17 surviv* OR survival [mh] OR mortalit* OR mortality [mh] OR mortality [sh] OR death* OR death [mh] OR os

#18 toxic* OR toxic actions [mh] OR "side effect*" OR "adverse effect*" OR "adverse event*" OR "adverse outcome*" OR "adverse reaction*" OR adverse effects [sh] OR complication* OR complications [sh]

#19 progress* OR disease progression [mh] OR progression-free survival [mh] OR pfs OR lpfs OR dpfs

#20 control* OR "stable disease*" OR response* OR regress* OR remission* OR remission induction [mh]

#21 "quality of life" OR quality of life [mh] OR qol OR hrqol

#22 "systemic therapy-free survival" OR "androgen deprivation-free survival" OR "androgen deprivation therapy-free survival" OR "ad-free survival" OR "adt-free survival" OR stfs OR adfs OR adtfs

#23 #17 OR #18 OR #19 OR #20 OR #21 OR #22

#24 #11 AND #16 AND #23

#25 randomized controlled trial [pt]

#26 controlled clinical trial [pt]

#27 randomized [tiab]

#28 placebo [tiab]

#29 drug therapy [sh]

#30 randomly [tiab]

#31 trial [tiab]

#32 groups [tiab]

#33 #25 OR #26 OR #27 OR #28 OR #29 OR #30 OR #31 OR #32

#34 animals [mh] NOT humans [mh]

#35 #33 NOT #34

#36 #24 AND #35

#37 systematic [sb]

#38 #24 AND #37

**Embase (through Embase.com)**

***Randomized trials***

No. Query

#1 prostat* OR 'prostate'/exp

#2 neoplasm* OR 'neoplasm'/exp

#3 tumor* OR tumour*

#4 malign*

#5 cancer*

#6 #2 OR #3 OR #4 OR #5

#7 relaps* OR 'relapse'/de OR recur* OR 'recurrent disease'/de OR 'tumor recurrence'/de OR 'cancer recurrence'/de

#8 metasta*

#9 #7 OR #8

#10 oligo*

#11 (#1 OR #6) AND #9 AND #10

#12 radiosurg* OR 'radiosurgery'/exp OR srs

#13 cyberkni* OR 'cyber kni*' OR 'cyberknife'/de

#14 (stereotactic OR stereotaxic) AND (radiotherap* OR radiation OR 'radiotherapy'/exp) OR 'stereotactic procedure'/exp OR srt OR sbrt

#15 ablative* AND (radiotherap* OR radiation OR 'radiotherapy'/exp) OR radioablati* OR sabr

#16 #12 OR #13 OR #14 OR #15

#17 surviv* OR 'survival'/exp OR mortalit* OR 'mortality'/exp OR death* OR 'death'/exp OR os

#18 toxic* OR 'toxicity'/exp OR 'side effect*' OR 'side effect'/exp OR 'adverse effect*' OR 'adverse event*' OR 'adverse outcome*' OR 'adverse reaction*' OR 'adverse event'/exp OR 'adverse outcome'/de OR complication* OR 'complication'/exp

#19 progress* OR 'disease exacerbation'/de OR 'tumor growth'/de OR 'cancer growth'/de OR 'progression-free survival' OR 'progression free survival'/exp OR pfs OR lpfs OR dpfs

#20 control* OR 'disease control'/exp OR 'cancer inhibition'/de OR 'metastasis inhibition'/de OR 'stable disease*' OR 'response*' OR regress* OR 'tumor regression'/de OR 'cancer regression'/de OR remission* OR 'remission'/de

#21 'quality of life' OR 'quality of life'/exp OR 'quality of life assessment'/exp OR qol OR hrqol

#22 'systemic therapy-free survival' OR 'androgen deprivation-free survival' OR 'androgen deprivation therapyfree survival' OR 'ad-free survival' OR 'adt-free survival' OR stfs OR adfs OR adtfs

#23 #17 OR #18 OR #19 OR #20 OR #21 OR #22

#24 #11 AND #16 AND #23

#25 'randomized controlled trial'/de

#26 'controlled clinical trial'/de

#27 random*:ti,ab

#28 'randomization'/de

#29 'intermethod comparison'/de

#30 placebo:ti,ab

#31 compare:ti OR compared:ti OR comparison:ti

#32 (evaluated:ab OR evaluate:ab OR evaluating:ab OR assessed:ab OR assess:ab) AND (compare:ab OR compared:ab OR comparing:ab OR comparison:ab)

#33 (open NEXT/1 label):ti,ab

#34 ((double OR single OR doubly OR singly) NEXT/1 (blind OR blinded OR blindly)):ti,ab

#35 'double blind procedure'/de

#36 (parallel NEXT/1 group*):ti,ab

#37 crossover:ti,ab OR 'cross over':ti,ab

#38 ((assign* OR match OR matched OR allocation) NEAR/6 (alternate OR group OR groups OR intervention OR interventions OR patient OR patients OR subject OR subjects OR participant OR participants)):ti,ab

#39 assigned:ti,ab OR allocated:ti,ab

#40 (controlled NEAR/8 (study OR design OR trial)):ti,ab

#41 volunteer:ti,ab OR volunteers:ti,ab

#42 'human experiment'/de

#43 trial:ti

#44 #25 OR #26 OR #27 OR #28 OR #29 OR #30 OR #31 OR #32 OR #33 OR #34 OR #35 OR #36 OR #37 OR #38 OR #39 OR #40 OR #41 OR #42 OR #43

#45 ((random* NEXT/1 sampl* NEAR/8 ('cross section*' OR questionnaire* OR survey OR surveys OR database OR databases)):ti,ab) NOT ('comparative study'/de OR 'controlled study'/de OR 'randomised controlled':ti,ab OR 'randomized controlled':ti,ab OR 'randomly assigned':ti,ab)

#46 'cross‐sectional study' NOT ('randomized controlled trial'/de OR 'controlled clinical study'/de OR 'controlled study'/de OR 'randomised controlled':ti,ab OR 'randomized controlled':ti,ab OR 'control group':ti,ab OR 'control groups':ti,ab)

#47 'case control*':ti,ab AND random*:ti,ab NOT ('randomised controlled':ti,ab OR 'randomized controlled':ti,ab)

#48 'systematic review':ti NOT (trial:ti OR study:ti)

#49 nonrandom*:ti,ab NOT random*:ti,ab

#50 'random field*':ti,ab

#51 ('random cluster' NEAR/4 sampl*):ti,ab

#52 review:ab AND review:it NOT trial:ti

#53 'we searched':ab AND (review:ti OR review:it)

#54 'update review':ab

#55 (databases NEAR/5 searched):ab

#56 (rat:ti OR rats:ti OR mouse:ti OR mice:ti OR swine:ti OR porcine:ti OR murine:ti OR sheep:ti OR lambs:ti OR pigs:ti OR piglets:ti OR rabbit:ti OR rabbits:ti OR cat:ti OR cats:ti OR dog:ti OR dogs:ti OR cattle:ti OR bovine:ti OR monkey:ti OR monkeys:ti OR trout:ti OR marmoset*:ti) AND 'animal experiment'/de

#57 'animal experiment'/de NOT ('human experiment'/de OR 'human'/de)

#58 #45 OR #46 OR #47 OR #48 OR #49 OR #50 OR #51 OR #52 OR #53 OR #54 OR #55 OR #56 OR #57

#59 #44 NOT #58

#60 #24 AND #59

***Systematic reviews***

No. Query

#1 prostat* OR 'prostate'/exp

#2 neoplasm* OR 'neoplasm'/exp

#3 tumor* OR tumour*

#4 malign*

#5 cancer*

#6 #2 OR #3 OR #4 OR #5

#7 relaps* OR 'relapse'/de OR recur* OR 'recurrent disease'/de OR 'tumor recurrence'/de OR 'cancer recurrence'/de

#8 metasta*

#9 #7 OR #8

#10 oligo*

#11 (#1 OR #6) AND #9 AND #10

#12 radiosurg* OR 'radiosurgery'/exp OR srs

#13 cyberkni* OR 'cyber kni*' OR 'cyberknife'/de

#14 (stereotactic OR stereotaxic) AND (radiotherap* OR radiation OR 'radiotherapy'/exp) OR 'stereotactic procedure'/exp OR srt OR sbrt

#15 ablative* AND (radiotherap* OR radiation OR 'radiotherapy'/exp) OR radioablati* OR sabr

#16 #12 OR #13 OR #14 OR #15

#17 surviv* OR 'survival'/exp OR mortalit* OR 'mortality'/exp OR death* OR 'death'/exp OR os

#18 toxic* OR 'toxicity'/exp OR 'side effect*' OR 'side effect'/exp OR 'adverse effect*' OR 'adverse event*' OR 'adverse outcome*' OR 'adverse reaction*' OR 'adverse event'/exp OR 'adverse outcome'/de OR complication* OR 'complication'/exp

#19 progress* OR 'disease exacerbation'/de OR 'tumor growth'/de OR 'cancer growth'/de OR 'progression-free survival' OR 'progression free survival'/exp OR pfs OR lpfs OR dpfs

#20 control* OR 'disease control'/exp OR 'cancer inhibition'/de OR 'metastasis inhibition'/de OR 'stable disease*' OR 'response*' OR regress* OR 'tumor regression'/de OR 'cancer regression'/de OR remission* OR 'remission'/de

#21 'quality of life' OR 'quality of life'/exp OR 'quality of life assessment'/exp OR qol OR hrqol

#22 'systemic therapy-free survival' OR 'androgen deprivation-free survival' OR 'androgen deprivation therapyfree survival' OR 'ad-free survival' OR 'adt-free survival' OR stfs OR adfs OR adtfs

#23 #17 OR #18 OR #19 OR #20 OR #21 OR #22

#24 #11 AND #16 AND #23

#25 'randomized controlled trial'/de

#26 'controlled clinical trial'/de

#27 random*:ti,ab

#28 'randomization'/de

#29 'intermethod comparison'/de

#30 placebo:ti,ab

#31 compare:ti OR compared:ti OR comparison:ti

#32 (evaluated:ab OR evaluate:ab OR evaluating:ab OR assessed:ab OR assess:ab) AND (compare:ab OR compared:ab OR comparing:ab OR comparison:ab)

#33 (open NEXT/1 label):ti,ab

#34 ((double OR single OR doubly OR singly) NEXT/1 (blind OR blinded OR blindly)):ti,ab

#35 'double blind procedure'/de

#36 (parallel NEXT/1 group*):ti,ab

#37 crossover:ti,ab OR 'cross over':ti,ab

#38 ((assign* OR match OR matched OR allocation) NEAR/6 (alternate OR group OR groups OR intervention OR interventions OR patient OR patients OR subject OR subjects OR participant OR participants)):ti,ab

#39 assigned:ti,ab OR allocated:ti,ab

#40 (controlled NEAR/8 (study OR design OR trial)):ti,ab

#41 volunteer:ti,ab OR volunteers:ti,ab

#42 'human experiment'/de

#43 trial:ti

#44 #25 OR #26 OR #27 OR #28 OR #29 OR #30 OR #31 OR #32 OR #33 OR #34 OR #35 OR #36 OR #37 OR #38 OR #39 OR #40 OR #41 OR #42 OR #43

#45 ((random* NEXT/1 sampl* NEAR/8 ('cross section*' OR questionnaire* OR survey OR surveys OR database OR databases)):ti,ab) NOT ('comparative study'/de OR 'controlled study'/de OR 'randomised controlled':ti,ab OR 'randomized controlled':ti,ab OR 'randomly assigned':ti,ab)

#46 'cross‐sectional study' NOT ('randomized controlled trial'/de OR 'controlled clinical study'/de OR 'controlled study'/de OR 'randomised controlled':ti,ab OR 'randomized controlled':ti,ab OR 'control group':ti,ab OR 'control groups':ti,ab)

#47 'case control*':ti,ab AND random*:ti,ab NOT ('randomised controlled':ti,ab OR 'randomized controlled':ti,ab)

#48 'systematic review':ti NOT (trial:ti OR study:ti)

#49 nonrandom*:ti,ab NOT random*:ti,ab

#50 'random field*':ti,ab

#51 ('random cluster' NEAR/4 sampl*):ti,ab

#52 review:ab AND review:it NOT trial:ti

#53 'we searched':ab AND (review:ti OR review:it)

#54 'update review':ab

#55 (databases NEAR/5 searched):ab

#56 (rat:ti OR rats:ti OR mouse:ti OR mice:ti OR swine:ti OR porcine:ti OR murine:ti OR sheep:ti OR lambs:ti OR pigs:ti OR piglets:ti OR rabbit:ti OR rabbits:ti OR cat:ti OR cats:ti OR dog:ti OR dogs:ti OR cattle:ti OR bovine:ti OR monkey:ti OR monkeys:ti OR trout:ti OR marmoset*:ti) AND 'animal experiment'/de

#57 'animal experiment'/de NOT ('human experiment'/de OR 'human'/de)

#58 #45 OR #46 OR #47 OR #48 OR #49 OR #50 OR #51 OR #52 OR #53 OR #54 OR #55 OR #56 OR #57

#59 #44 NOT #58

#60 #24 AND #59

#61 'systematic review'/de

#62 #24 AND #61

**Cumulative Index to Nursing and Allied Health Literature Complete (through EBSCOhost)**

***Randomized trials***

# Query

S1 TX prostat* OR MH prostate

S2 TX neoplasm* OR MH neoplasms+

S3 TX tumor* OR TX tumour*

S4 TX malign*

S5 TX cancer*

S6 S2 OR S3 OR S4 OR S5

S7 TX relaps* OR TX recur* OR MH recurrence

S8 TX metasta*

S9 S7 OR S8

S10 TX oligo*

S11 (S1 OR S6) AND S9 AND S10

S12 TX radiosurg* OR MH radiosurgery OR TX srs

S13 TX cyberkni* OR TX ”cyber kni*”

S14 ((TX stereotactic* OR TX stereotaxic*) AND (TX radiotherap* OR TX radiation OR MH radiotherapy+)) OR MH stereotaxic techniques+ OR TX srt OR TX sbrt

S15 (TX ablative* AND (TX radiotherap* OR TX radiation OR MH radiotherapy+)) OR TX radioablati* OR TX sabr

S16 S12 OR S13 OR S14 OR S15

S17 TX surviv* OR MH survival OR TX mortalit* OR MH mortality+ OR MW “MO” OR TX death* OR MH death+ OR TX os

S18 TX toxic* OR TX “side effect*” OR TX “adverse effect*” OR TX “adverse event*” OR TX “adverse outcome*” OR TX “adverse reaction*” OR MH adverse health care event+ OR MW “AE” OR TX complication*

S19 TX progress* OR MH disease exacerbation OR MH disease progression+ OR TX pfs OR TX lpfs OR TX dpfs

S20 TX control* OR TX “stable disease*” OR TX response* OR TX regress* OR TX remission* OR MH disease remission

S21 TX “quality of life” OR MH quality of life+ OR TX qol OR TX hrqol

S22 TX “systemic therapy-free survival” OR TX “androgen deprivation-free survival” OR TX “androgen deprivation therapy-free survival” OR TX “ad-free survival” OR TX “adt-free survival” OR TX stfs OR TX adfs OR TX adtfs

S23 S17 OR S18 OR S19 OR S20 OR S21 OR S22

S24 S11 AND S16 AND S23

S25 (MH "Clinical Trials+")

S26 PT Clinical trial

S27 TX clinic* n1 trial*

S28 TX ( (singl* n1 blind*) or (singl* n1 mask*) ) or TX ( (doubl* n1 blind*) or (doubl* n1 mask*) ) or TX ( (tripl* n1 blind*) or (tripl* n1 mask*) ) or TX ( (trebl* n1 blind*) or (trebl* n1 mask*) )

S29 TX randomi* control* trial*

S30 (MH "Random Assignment")

S31 TX random* allocat*

S32 TX placebo*

S33 (MH "Placebos")

S34 (MH "Quantitative Studies")

S35 TX allocat* random*

S36 S25 OR S26 OR S27 OR S28 OR S29 OR S30 OR S31 OR S32 OR S33 OR S34 OR S35

S37 S24 AND S36

***Systematic reviews***

# Query

S1 TX prostat* OR MH prostate

S2 TX neoplasm* OR MH neoplasms+

S3 TX tumor* OR TX tumour*

S4 TX malign*

S5 TX cancer*

S6 S2 OR S3 OR S4 OR S5

S7 TX relaps* OR TX recur* OR MH recurrence

S8 TX metasta*

S9 S7 OR S8

S10 TX oligo*

S11 (S1 OR S6) AND S9 AND S10

S12 TX radiosurg* OR MH radiosurgery OR TX srs

S13 TX cyberkni* OR TX ”cyber kni*”

S14 ((TX stereotactic* OR TX stereotaxic*) AND (TX radiotherap* OR TX radiation OR MH radiotherapy+)) OR MH stereotaxic techniques+ OR TX srt OR TX sbrt

S15 (TX ablative* AND (TX radiotherap* OR TX radiation OR MH radiotherapy+)) OR TX radioablati* OR TX sabr

S16 S12 OR S13 OR S14 OR S15

S17 TX surviv* OR MH survival OR TX mortalit* OR MH mortality+ OR MW “MO” OR TX death* OR MH death+ OR TX os

S18 TX toxic* OR TX “side effect*” OR TX “adverse effect*” OR TX “adverse event*” OR TX “adverse outcome*” OR TX “adverse reaction*” OR MH adverse health care event+ OR MW “AE” OR TX complication*

S19 TX progress* OR MH disease exacerbation OR MH disease progression+ OR TX pfs OR TX lpfs OR TX dpfs

S20 TX control* OR TX “stable disease*” OR TX response* OR TX regress* OR TX remission* OR MH disease remission

S21 TX “quality of life” OR MH quality of life+ OR TX qol OR TX hrqol

S22 TX “systemic therapy-free survival” OR TX “androgen deprivation-free survival” OR TX “androgen deprivation therapy-free survival” OR TX “ad-free survival” OR TX “adt-free survival” OR TX stfs OR TX adfs OR TX adtfs

S23 S17 OR S18 OR S19 OR S20 OR S21 OR S22

S24 S11 AND S16 AND S23

S25 (MH "Clinical Trials+")

S26 PT Clinical trial

S27 TX clinic* n1 trial*

S28 TX ( (singl* n1 blind*) or (singl* n1 mask*) ) or TX ( (doubl* n1 blind*) or (doubl* n1 mask*) ) or TX ( (tripl* n1 blind*) or (tripl* n1 mask*) ) or TX ( (trebl* n1 blind*) or (trebl* n1 mask*) )

S29 TX randomi* control* trial*

S30 (MH "Random Assignment")

S31 TX random* allocat*

S32 TX placebo*

S33 (MH "Placebos")

S34 (MH "Quantitative Studies")

S35 TX allocat* random*

S36 S25 OR S26 OR S27 OR S28 OR S29 OR S30 OR S31 OR S32 OR S33 OR S34 OR S35

S37 S24 AND S36

S38 PT systematic review OR MH systematic review OR TI (systematic* AND review*) OR AB (systematic* AND review*)

S39 S24 AND S38

**Cochrane Central Register of Controlled Trials and Cochrane Database of Systematic Reviews (through Cochrane Library)**

ID Search

#1 (prostat*)

#2 MeSH descriptor: [Prostate] explode all trees

#3 #1 OR #2

#4 (neoplasm*)

#5 MeSH descriptor: [Neoplasms] explode all trees

#6 (tumor* OR tumour*)

#7 (malign*)

#8 (cancer*)

#9 (5-#8)

#10 (relaps* OR recur*)

#11 MeSH descriptor: [Recurrence] explode all trees

#12 (metasta*)

#13 {OR #10-#12}

#14 (oligo*)

#15 (#3 OR #9) AND #13 AND #14

#16 (radiosurg* OR srs)

#17 MeSH descriptor: [Radiosurgery] explode all trees

#18 (cyberkni* OR ”cyber kni*”)

#19 MeSH descriptor: [Radiotherapy] explode all trees

#20 (((stereotactic* OR stereotaxic*) AND (radiotherap* OR radiation OR #19)) OR srt OR sbrt)

#21 MeSH descriptor: [Stereotaxic Techniques] explode all trees

#22 ((ablative* AND (radiotherap* OR radiation OR #19)) OR radioablati* OR sabr)

#23 {OR #16-#18, #20-#22}

#24 (surviv* OR mortalit* OR death* OR os)

#25 MeSH descriptor: [Survival] explode all trees

#26 MeSH descriptor: [Mortality] explode all trees

#27 MeSH descriptor: [] explode all trees and with qualifier(s): [mortality - MO]

#28 MeSH descriptor: [Death] explode all trees

#29 (toxic* OR “side effect*” OR “adverse effect*” OR “adverse event*” OR “adverse outcome*” OR “adverse reaction*” OR complication*)

#30 MeSH descriptor: [Toxic Actions] explode all trees

#31 MeSH descriptor: [] explode all trees and with qualifier(s): [adverse effects - AE]

#32 MeSH descriptor: [] explode all trees and with qualifier(s): [complications - CO]

#33 (progress* OR pfs OR lpfs OR dpfs)

#34 MeSH descriptor: [Disease Progression] explode all trees

#35 MeSH descriptor: [Progression-Free Survival] explode all trees

#36 (control* OR “stable disease*” OR response* OR regress* OR remission*)

#37 MeSH descriptor: [Remission Induction] explode all trees

#38 (“quality of life” OR qol OR hrqol)

#39 MeSH descriptor: [Quality of Life] explode all trees

#40 (“systemic therapy-free survival” OR “androgen deprivation-free survival” OR “androgen deprivation therapy-free survival” OR “ad-free survival” OR “adt-free survival” OR stfs OR adfs OR adtfs)

#41 {OR #24-#40}

#42 {AND #15, #23, #41}

**ClinicalTrials.gov**

***Advanced search interface***

*“Other terms” field*

(metastasis OR metastatic OR oligometastasis OR oligometastatic) AND (radiosurgery OR srs OR cyberknife OR ((stereotactic OR stereotaxic OR ablative) AND radiation therapy) OR srt OR sbrt OR radioablation OR radioablative OR sabr)

*Filters*

Age Group: Adult (18–64), Older Adult (65+)

**International Clinical Trials Registry Platform**

***Standard search interface***

metasta* AND radiosurg* OR metasta* AND srs OR metasta* AND cyber knife OR metasta* AND cyberknife OR metasta* AND stereotactic AND radiation therapy OR metasta* AND stereotaxic AND radiation therapy OR metasta* AND srt OR metasta* AND sbrt OR metasta* AND ablative AND radiation therapy OR metasta* AND radioablati* OR metasta* AND sabr OR oligo* AND radiosurg* OR oligo* AND srs OR oligo* AND cyber knife OR oligo* AND cyberknife OR oligo* AND stereotactic AND radiation therapy OR oligo* AND stereotaxic AND radiation therapy OR oligo* AND srt OR oligo* AND sbrt OR oligo* AND ablative AND radiation therapy OR oligo* AND radioablati* OR oligo* AND sabr
