# Supplementary material for: Stereotactic body radiotherapy as metastasis-directed therapy in oligometastatic prostate cancer: a systematic review and meta-analysis of randomized controlled trials
Source: Radiat Oncol. 2024 Dec 17;19:173. doi: 10.1186/s13014-024-02559-7 (PMC11654405; doi:10.1186/s13014-024-02559-7)
Supplement: Supplementary file 2 — Additional file 2. [file 13014_2024_2559_MOESM2_ESM.doc]

**Additional file 2.** Deduplication strategy

1. If all available information on author, year, title, journal, number and pages suggest it is the same record, it was regarded as a duplicate record.
2. If the records differ on author, journal title, volume and/or pages, the Digital Object Identifier numbers were compared. If identical, the reports were regarded as duplicates.
3. If the records differ on author, journal title, volume and/or pages, the abstracts were compared. If identical, the reports were regarded as duplicates.
4. If multiple records of the same trial were found to be added to the Cochrane Central Register of Controlled Trials (CENTRAL) at different times with limited or no information on journal title, volume, and pages, the records were regarded as duplicates.
5. If multiple conference abstracts were found to report on the same study, the one with the longest follow-up, the latest one or the one with most information was chosen and the others regarded as duplicates.
6. If several records were deemed to communicate on the same study, the full text record was chosen and the others (e.g., conference abstracts, protocols) treated as duplicates.
7. If we find conference abstracts communicating on a protocol and the original protocol, we will regard the conference abstract as a duplicate.
8. If we find only a conference abstract reporting on a protocol of a study, but no protocol, the abstract will be treated as a protocol.
9. If multiple protocols were found, we picked the one last updated or published and regarded the others as duplicates.
