# Supplementary material for: Stereotactic body radiotherapy as metastasis-directed therapy in oligometastatic prostate cancer: a systematic review and meta-analysis of randomized controlled trials
Source: Radiat Oncol. 2024 Dec 17;19:173. doi: 10.1186/s13014-024-02559-7 (PMC11654405; doi:10.1186/s13014-024-02559-7)
Supplement: Supplementary file 3 — Additional file 3. [file 13014_2024_2559_MOESM3_ESM.doc]

**Additional file 3.** Order of importance of exclusion criteria

1. Not informing on original research
2. Ineligible study design
3. Ineligible participants
4. Ineligible intervention
5. Ineligible control group
6. Ineligible outcomes
7. Retracted study
8. Insufficient information for qualitative synthesis
9. Insufficient information for quantitative synthesis
