# Supplementary material for: Stereotactic body radiotherapy as metastasis-directed therapy in oligometastatic prostate cancer: a systematic review and meta-analysis of randomized controlled trials
Source: Radiat Oncol. 2024 Dec 17;19:173. doi: 10.1186/s13014-024-02559-7 (PMC11654405; doi:10.1186/s13014-024-02559-7)
Supplement: Supplementary file 4 — Additional file 4. [file 13014_2024_2559_MOESM4_ESM.doc]

**Additional file 4.** Characteristics of ongoing studies

**Stereotactic Body Radiotherapy for Extra-cranial Oligorecurrent Tumor: Randomized Phase II Clinical Trial [1]**

| **Registration number** | NCT02410187 |
| --- | --- |
| **Methods** | RCT with parallel assignment. Phase II. Single-center. |
| **Participants** | Recruitment target: 1 participant  Any malignancy except lymphoma, myeloma, and germ cell tumors  1–5 metastases  Controlled primary tumor  No recurring metastases after previous RT |
| **Intervention** | SOC + SBRT |
| **Control** | SOC |
| **Outcomes** | Primary:  Disease PFS rate  Secondary:  Overall survival rate, LC rate, Number of participants with radiation-induced acute or late toxicity |
| **Starting date** | March 2016 |
| **Estimated completion** | March 2020 (Last update March 2016) |
| **Notes** |  |

**The Role of Stereotactic Body Radiotherapy in the Management of Castration-Resistant Prostate Cancer With Oligometastases: An Adaptive Phase II/III Randomized Trial (PCS IX) [2]**

| **Registration number** | NCT02685397 |
| --- | --- |
| **Methods** | RCT with parallel assignment. Phase II/III. Multicenter. |
| **Participants** | Recruitment target: 130 participants  PCa  1–5 metastases with ≤4 per organ system (excluding brain and liver)  Ongoing ADT with progressive disease  No previous cytotoxic chemotherapy for PCa  Previously treated primary tumor |
| **Intervention** | ADT + enzalutamide + SBRT |
| **Control** | ADT + enzalutamide |
| **Outcomes** | Primary:  Radiographic PFS  Secondary:  QoL, Toxicity, PCa-specific survival, Time to skeletal-related event, Overall survival, LC, Time to systemic antineoplastic therapy, PSA response, Predictive value of biomarkers |
| **Starting date** | October 2016 |
| **Estimated completion** | August 2041 |
| **Notes** |  |

**Extracranial Stereotactic Body Radiation Therapy (SBRT) Added to Standard Treatment Versus Standard Treatment Alone in Solid Tumors Patients With Between 1 and 5 Bone-only Metastases (STEREO-OS) [3]**

| **Registration number** | NCT03143322 |
| --- | --- |
| **Methods** | RCT with parallel assignment. Phase III. Multicenter. |
| **Participants** | Recruitment target: 196 participants  PCa, breast cancer, or non-small cell lung cancer  1–3 bone metastases as defined by NaF PET ± spinal MRI  No previous systemic therapy for metachronous metastatic disease except hormonal therapy initiated before enrollment  Synchronous or metachronous metastatic disease  Primary tumor previously treated or accessible to curative-intent treatment if locoregional recurrence |
| **Intervention** | SOC + SBRT |
| **Control** | SOC |
| **Outcomes** | Primary:  PFS  Secondary:  Bone PFS, LC, Cancer-specific survival, Overall survival, SBRT toxicities, Patient’s QoL, Pain score, Cost utility |
| **Starting date** | January 2018 |
| **Estimated completion** | July 2027 |
| **Notes** | Published protocol [4] |

**A Randomized Phase III Trial of Stereotactic Ablative Radiotherapy for the Comprehensive Treatment of 4-10 Oligometastatic Tumors (SABR-COMET-10) [5]**

| **Registration number** | NCT03721341 |
| --- | --- |
| **Methods** | RCT with parallel assignment. Phase III. Multicenter. |
| **Participants** | Recruitment: 204 participants  Any malignancy  4–10 metastases  Controlled primary tumor |
| **Intervention** | SOC + SBRT |
| **Control** | SOC |
| **Outcomes** | Primary:  Overall survival  Secondary:  PFS, Time from randomization to development of new metastatic lesions, QoL as measured by FACT-G questionnaire, QoL as measured by EQ-5D-5L questionnaire, Toxicity as measured by CTCAE v4.0, Overall survival at midpoint of study |
| **Starting date** | February 2019 |
| **Estimated completion** | January 2029 |
| **Notes** | Published protocol [6] |

**Phase III Randomized Controlled Trial and Economic Evaluation of Stereotactic Ablative Radiotherapy for Comprehensive Treatment of Oligometastatic (1-3 Metastases) Cancer (SABR-COMET-3) [7]**

| **Registration number** | NCT03862911 |
| --- | --- |
| **Methods** | RCT with parallel assignment. Phase III. Multicenter. |
| **Participants** | Recruitment target: 330 participants  Any malignancy  1–3 current metastases (≤8 metastases during course of disease) on CT and bone scan or PET-CT  Controlled primary tumor |
| **Intervention** | SOC + SBRT |
| **Control** | SOC |
| **Outcomes** | Primary:  Overall survival  Secondary:  Side effects, PFS, Patient-reported QoL, Health-related QoL questionnaire, Resource utilization, Correlation between candidate biomarkers of oligometastatic disease (blood-derived) and oncologic outcomes |
| **Starting date** | November 2019 |
| **Estimated completion** | December 2028 |
| **Notes** | Published protocol [8] |

**A Randomized, Phase II Study of Apalutamide +/- Stereotactic Body Radiotherapy (SBRT) in Castration-Resistant Prostate Cancer Patients With Oligometastatic Disease on PSMA-PET Imaging (PILLAR) [9]**

| **Registration number** | NCT03503344 |
| --- | --- |
| **Methods** | RCT with parallel assignment. Phase II. Single-center. |
| **Participants** | Recruitment target: 26 participants  PCa  1–5 radiation fields with PET-avid lesions on PSMA PET-CT  Progressive CRPC  No previous systemic therapy for CRPC |
| **Intervention** | ADT + apalutamide + SBRT |
| **Control** | ADT + apalutamide |
| **Outcomes** | Primary:  Proportion of patients with undetectable serum PSA  Secondary:  Median time to PSA progression, Frequency of treatment-related AEs |
| **Starting date** | December 2019 |
| **Estimated completion** | January 2027 |
| **Notes** |  |

**Prostate-cancer Treatment Using Stereotactic Radiotherapy for Oligometastases Ablation in Hormone-sensitive Patients - a GETUG-AFU Phase III Randomized Controlled Trial (PRESTO) [10]**

| **Registration number** | NCT04115007 |
| --- | --- |
| **Methods** | RCT with parallel assignment. Phase III. Multicenter. |
| **Participants** | Recruitment target: 350 participants  PCa  1–5 metastases (≥1 bone or lung ± lymph nodes) on choline or PSMA PET-CT or whole-body MRI  Hormone-sensitive disease  No previous MDT |
| **Intervention** | SOC + SBRT |
| **Control** | SOC |
| **Outcomes** | Primary:  CRPC-free survival  Secondary:  Overall survival, PCa-specific survival, Time to castration resistance, Time to next symptomatic skeletal event, Time to next symptomatic skeletal event at the treated metastatic bone sites, Time to use of intermittent hormonal therapy, Duration of intermittent hormonal therapy, Time to secondary treatments (local or systemic), Acute and late toxicity of stereotactic RT of oligometastases, Severity of pain during treatment, EQ-5D-3L questionnaire, EPIC short form, Cost-effectiveness analysis of the proposed therapeutic strategy |
| **Starting date** | June 2020 |
| **Estimated completion** | June 2028 |
| **Notes** |  |

**Intermittent Androgen Deprivation Therapy With or Without Stereotactic Body Radiotherapy for Molecularly Identified Hormone Sensitive Oligometastatic Prostate Cancer: A Randomized Feasibility Study (iSTOP) [11]**

| **Registration number** | NCT04619069 |
| --- | --- |
| **Methods** | RCT with parallel assignment. Phase I/II. Single-center. |
| **Participants** | Recruitment target: 30 participants  PCa  1–3 metastases on PSMA PET-CT not visible on bone scan, CT, and/or MRI  Biochemical recurrence  Synchronous or metachronous metastatic disease  Hormone-sensitive disease  Controlled primary tumor |
| **Intervention** | Intermittent hormone therapy + SBRT |
| **Control** | Intermittent hormone therapy |
| **Outcomes** | Primary:  Proportion of eligible patients who enroll onto the study  Secondary:  Side effects and effectiveness |
| **Starting date** | October 2020 |
| **Estimated completion** | October 2027 |
| **Notes** |  |

**Veterans Affairs Seamless Phase II/III Randomized Trial of STAndard Systemic theRapy With or Without PET-directed Local Therapy for OligoRecurrenT Prostate Cancer (VA STARPORT) [12]**

| **Registration number** | NCT04787744 |
| --- | --- |
| **Methods** | RCT with parallel assignment. Phase II/III. Multicenter. |
| **Participants** | Recruitment target: 464 participants  PCa  1–10 metastases  Hormone-sensitive disease  Eligible for PET-directed local therapy |
| **Intervention** | SOC ± local salvage therapy + MDT (e.g., SBRT) |
| **Control** | SOC |
| **Outcomes** | Primary:  CRPC-free survival  Secondary:  Radiographic PFS, Clinical PFS, Freedom from index lesion progression, New metastasis-free survival, PCa-specific survival, Overall survival, CTCAE v5.0 toxicity, Patient-reported quality of life measured by the EORTC QLQ-C30 3.0, EPIC-26, Patient-reported health-related QoL measured by the EQ-5D-5L |
| **Starting date** | July 2021 |
| **Estimated completion** | December 2025 |
| **Notes** |  |

**Metastasis Directed Stereotactic Body Radiotherapy for Oligo Metastatic Hormone Sensitive Prostate Cancer (METRO) [13]**

| **Registration number** | NCT04983095 |
| --- | --- |
| **Methods** | RCT with parallel assignment. Phase III. Multicenter. |
| **Participants** | Recruitment target: 118 participants  PCa  1–3 bone or extra-pelvic nodal metastases on PSMA PET-CT  *De novo* metastatic disease or biochemical relapse after definitive prostate RT or radical prostatectomy |
| **Intervention** | ADT ± RT to prostate + SBRT |
| **Control** | ADT ± RT to prostate |
| **Outcomes** | Primary:  Failure-free survival  Secondary:  Number of participants with treatment-related adverse events as assessed by CTCAE v5, Number of participants with treatment-related serious adverse events as assessed by CTCAE v5, Outcome prediction, Patient reported quality of life assessed by EORTC QLQ-30, Overall survival, CRPC |
| **Starting date** | October 2021 |
| **Estimated completion** | December 2031 |
| **Notes** |  |

**TArgeted STEreotactic Radiotherapy for Oligorecurrent PROstate Cancer - a Randomized Controlled Pilot Trial (TASTEPRO) [14]**

| **Registration number** | NCT05067660 |
| --- | --- |
| **Methods** | RCT with parallel assignment. Phase NA. Single-center. |
| **Participants** | Recruitment target: 40 participants  PCa  Biochemical recurrence after radical prostatectomy  1–3 lymph node metastases (N1 or M1a) on PSMA PET-CT  Planned salvage RT without ADT |
| **Intervention** | SBRT |
| **Control** | SOC salvage RT with conventional fractionation ± boost |
| **Outcomes** | Primary:  Undetectable serum PSA after salvage RT  Secondary:  Time from salvage RT until PSA increase, Initiation of ADT or bicalutamide, Urinary and sexual symptoms after salvage RT, Self-reported post-treatment QoL, Serum thymidine kinase 1, Amount of plasma ctDNA prior to radiation therapy as predictor of disease burden (number and size of detected lesions) discovered in the PSMA PET-CT, Inter-reader agreement of PSMA PET-CT interpretation |
| **Starting date** | January 2022 |
| **Estimated completion** | January 2025 |
| **Notes** |  |

**Androgen Deprivation Therapy (ADT) Versus Stereotactic Body Radiotherapy (SBRT) for Oligometastatic Prostate Cancer: A Prospective Randomized Control Clinical Trial [15]**

| **Registration number** | NCT04599686 |
| --- | --- |
| **Methods** | RCT with parallel assignment. Phase NI. Single-center. |
| **Participants** | Recruitment target: 100 participants  PCa  1–3 bone or lymph node metastases (up to 5 if in the same area) on PSMA PET-CT  Previous treatment of the prostate with curative intent  No previous or ongoing systemic therapy for metastatic disease  No previous MDT |
| **Intervention** | SBRT |
| **Control** | ADT |
| **Outcomes** | Primary:  1-year ADT-free survival of the experimental group  The probability of RT-related toxicity  The time from inception of the study to CRPC    Secondary:  1-year efficacy biochemical PFS, 1-year local PFS, 1-year distant metastasis-free survival |
| **Starting date** | September 2022 |
| **Estimated completion** | October 2025 |
| **Notes** | Published protocol [16] |

**Phase 2 Randomized Total Eradication of Metastatic Lesions Following Definitive Radiation to the Prostate in de Novo oligometaStatic Prostate Cancer Trial (TERPS) [17]**

| **Registration number** | NCT05223803 |
| --- | --- |
| **Methods** | RCT with parallel assignment. Phase II. Multicenter. |
| **Participants** | Recruitment target: 122 participants  PCa  1–3 bone or soft tissue metastases (≥1 bone) on imaging or ≤5 on fluciclovine, choline, or PSMA PET-CT developed during the last 6 months  Hormone-sensitive disease |
| **Intervention** | SOC + SBRT |
| **Control** | SOC |
| **Outcomes** | Primary:  2-year FFS  Secondary:  To determine the number of participants with toxicities related to SABR MDT, To determine local control at 12 months after SABR, To assess time to progression, Quality of life assessed through EPIC tool |
| **Starting date** | October 2022 |
| **Estimated completion** | July 2027 |
| **Notes** |  |

**PRostate Only radiotherapy versus radiotherapy to all Sites in Oligo-metastatic prostate cancer - A phase II randomized open label study (PROS-O) [18]**

| **Registration number** | CTRI/2022/10/046239 |
| --- | --- |
| **Methods** | RCT with parallel assignment. Phase II. |
| **Participants** | Recruitment target: 42 participants  PCa  1–5 metastases  No previous RT to the prostate |
| **Intervention** | RT to prostate + SBRT |
| **Control** | RT to prostate |
| **Outcomes** | Primary:  12-month PFS  Secondary:  Overall survival, Acute toxicity |
| **Starting date** | October 2022 |
| **Estimated completion** | NI |
| **Notes** |  |

**Phase III Study of Stereotactic Body Radiation Therapy (SBRT) Plus Standard of Care in Castration Sensitive Oligometastatic Prostate Cancer Patients (START-MET) [19]**

| **Registration number** | NCT05209243 |
| --- | --- |
| **Methods** | RCT with parallel assignment. Phase III. Multicenter. |
| **Participants** | Recruitment target: 266 participants  PCa  ≤3 bone or lymph node metastases on CT and bone scan and ≤5 on choline or PSMA PET-CT  Hormone-sensitive disease  No previous treatment with docetaxel, second-generation androgen receptor inhibitor, or bone antiresorptive therapy |
| **Intervention** | SOC + SBRT |
| **Control** | SOC |
| **Outcomes** | Primary:  Radiological PFS  Secondary:  Overall survival, Time to cytotoxic chemotherapy, Time to PSA progression, Local control, Time to castration resistance, Time to skeletal-related event, Quality of life (FACT-P), Safety profile, Pain (BPI) |
| **Starting date** | January 2023 |
| **Estimated completion** | January 2027 |
| **Notes** |  |

**APalutamide and stEReotactic Body Radiation Therapy for Low Burden Metastatic Hormone senSItive Prostate Cancer, a rANdomized Trial (PERSIAN) [20]**

| **Registration number** | NCT05717660 |
| --- | --- |
| **Methods** | RCT with parallel assignment. Phase II. Multicenter. |
| **Participants** | Recruitment target: 180 participants  PCa  1–5 non-visceral metastases  Recurrence after curative treatment  Hormone-sensitive disease  Controlled primary tumor |
| **Intervention** | ADT + apalutamide + SBRT |
| **Control** | ADT + apalutamide |
| **Outcomes** | Primary:  Complete biochemical response  Secondary:  Freedom from biochemical progression, Freedom from radiological progression, Rate of adverse events, Overall survival, Cancer-specific survival, Health-related QoL |
| **Starting date** | March 2023 |
| **Estimated completion** | March 2025 |
| **Notes** | Published protocol [21] |

**A Randomized Phase III Trial of Stereotactic Ablative Radiotherapy for Patients With Up to 10 Oligometastases and a Synchronous Primary Tumor** **(SABR-SYNC) [22]**

| **Registration number** | NCT05717166 |
| --- | --- |
| **Methods** | RCT with parallel assignment. Phase III. Multicenter. |
| **Participants** | Recruitment target: 180 participants  Any malignancy  Metastases detectable on imaging  1–10 metastases  Primary tumor present |
| **Intervention** | SOC + local treatment to prostate + MDT (e.g., SBRT) |
| **Control** | SOC |
| **Outcomes** | Primary:  Overall survival  Secondary:  QoL assessed with FACT-G, QoL assessed with EQ-5D-5L, Toxicity assessed by CTCAE v5 for each organ treated, Time to next systemic therapy, Receipt of additional radiation during follow-up |
| **Starting date** | October 2023 |
| **Estimated completion** | April 2029 |
| **Notes** |  |

**Studying Treatments in Patients Receiving Androgen Deprivation Therapy (ADT) and Androgen Receptor Signalling Inhibitors (ARSI) for Metastatic Prostate Cancer: Evaluation of Drug and Radiation Efficacy: A 2nd Multi-arm Multi-stage Randomised Controlled Trial (STAMPEDE2) [23]**

| **Registration number** | NCT06320067 |
| --- | --- |
| **Methods** | RCT with parallel assignment. Phase III. Multicenter. |
| **Participants** | Recruitment target: 2476 participants  PCa  1–5 bone and/or non-regional lymph node metastases on CT, bone scan, or MRI  *De novo* metastatic disease  Hormone-sensitive disease |
| **Intervention** | SOC + SBRT |
| **Control** | SOC |
| **Outcomes** | Primary:  Radiographic PFS  Overall survival  Secondary:  Failure-free survival, PCa-specific survival, Safety through reporting of SAEs, Toxicity using CTCAE classification, Compliance with randomized allocation, EQ-5D-5L questionnaire for QoL and cost-effectiveness assessment |
| **Starting date** | April 2024 |
| **Estimated completion** | March 2034 |
| **Notes** |  |

Participants described according to eligibility criteria relevant to this review.

Abbreviations: ADT: androgen deprivation therapy; AE: adverse event; BPI: Brief Pain Inventory; CRPC: castration-resistant prostate cancer; CT: computed tomography; CTCAE: Common Terminology Criteria for Adverse Events; ctDNA: circulating tumor DNA; EORTC: European Organization for Research and Treatment of Cancer; EPIC: Expanded Prostate Cancer Index Composite; EQ-5D: EuroQol 5-Dimension; EQ-5D-3L: 3-level version of EQ-5D; EQ-5D-5L: 5-level version of EQ-5D; FACT-G: Functional Assessment of Cancer Therapy-General; FACT-P: Functional Assessment of Cancer Therapy-Prostate; LC: local control; M: metastasis; MDT: metastasis-directed therapy; MRI: magnetic resonance imaging; N: node; NA: not applicable; NaF: sodium fluoride; NI: no information; PCa: prostate cancer; PET: positron emission tomography; PFS: progression-free survival; PSA: prostate-specific antigen; PSMA: prostate-specific membrane antigen; QLQ-C30: Quality of Life Questionnaire-Core 30; QoL: quality of life; RCT: randomized controlled trial; RT: radiotherapy; SABR: stereotactic ablative radiotherapy; SBRT: stereotactic body radiotherapy; SOC: standard of care; v: version

***References***

1. ClinicalTrials.gov: SBRT for extra-cranial oligorecurrent tumor (NCT02410187). https://clinicaltrials.gov/show/NCT02410187 (2015). Accessed 29 Mar 2024.

2. ClinicalTrials.gov: The role of stereotactic body radiotherapy in the management of castration-resistant prostate cancer with oligometastases: an adaptive phase II/III randomized trial (PCS IX) (NCT02685397). https://clinicaltrials.gov/show/NCT02685397 (2016). Accessed 23 Mar 2024.

3. ClinicalTrials.gov: Standard treatment +/- SBRT in solid tumors patients with between 1 and 3 bone-only metastases (STEREO-OS) (NCT03143322). https://clinicaltrials.gov/show/NCT03143322 (2017). Accessed 29 Mar 2024.

4. Thureau S, Marchesi V, Vieillard MH, Perrier L, Lisbona A, Leheurteur M, et al. Efficacy of extracranial stereotactic body radiation therapy (SBRT) added to standard treatment in patients with solid tumors (breast, prostate and non-small cell lung cancer) with up to 3 bone-only metastases: study protocol for a randomised phase III trial (STEREO-OS). BMC Cancer. 2021;21:117.

5. ClinicalTrials.gov: Stereotactic ablative radiotherapy for comprehensive treatment of 4-10 oligometastatic tumors (SABR-COMET-10) (NCT03721341). https://clinicaltrials.gov/show/NCT03721341 (2018). Accessed 26 Mar 2024.

6. Palma DA, Olson R, Harrow S, Correa RJM, Schneiders F, Haasbeek CJA, et al. Stereotactic ablative radiotherapy for the comprehensive treatment of 4-10 oligometastatic tumors (SABR-COMET-10): study protocol for a randomized phase III trial. BMC Cancer. 2019;19:816.

7. ClinicalTrials.gov: Phase III randomized controlled trial and economic evaluation of stereotactic ablative radiotherapy for comprehensive treatment of oligometastatic (1-3 metastases) cancer (SABR-COMET-3) (NCT03862911). https://clinicaltrials.gov/show/NCT03862911 (2019). Accessed 25 Mar 2024.

8. Olson R, Mathews L, Liu M, Schellenberg D, Mou B, Berrang T, et al. Stereotactic ablative radiotherapy for the comprehensive treatment of 1-3 oligometastatic tumors (SABR-COMET-3): study protocol for a randomized phase III trial. BMC Cancer. 2020;20:380.

9. ClinicalTrials.gov: A randomized, phase II study of apalutamide +/- stereotactic body radiotherapy (SBRT) in castration-resistant prostate cancer patients with oligometastatic disease on PSMA-pet imaging (PILLAR) (NCT03503344). https://clinicaltrials.gov/show/NCT03503344 (2018). Accessed 24 Mar 2024.

10. ClinicalTrials.gov: Prostate-cancer treatment using stereotactic radiotherapy for oligometastases ablation in hormone-sensitive patients - a GETUG-AFU phase III randomized controlled trial (PRESTO) (NCT04115007). https://clinicaltrials.gov/show/NCT04115007 (2019). Accessed 25 Mar 2024.

11. ClinicalTrials.gov: Intermittent androgen deprivation therapy with or without stereotactic body radiotherapy for molecularly identified hormone sensitive oligometastatic prostate cancer: a randomized feasibility study (NCT04619069). https://clinicaltrials.gov/show/NCT04619069 (2020). Accessed 23 Mar 2024.

12. ClinicalTrials.gov: Veterans affairs seamless phase II/III randomized trial of standard systemic therapy with or without PET-directed local therapy for oligorecurrent prostate cancer (VA STARPORT) (NCT04787744). https://clinicaltrials.gov/study/NCT04787744 (2021). Accessed 31 Mar 2024.

13. ClinicalTrials.gov: Metastasis directed stereotactic body radiotherapy for oligo metastatic hormone sensitive prostate cancer (METRO) (NCT04983095). https://clinicaltrials.gov/study/NCT04983095 (2021). Accessed 26 Mar 2024.

14. ClinicalTrials.gov: Targeted stereotactic radiotherapy for oligorecurrent prostate cancer - a randomized controlled pilot trial (TASTEPRO) (NCT05067660). https://clinicaltrials.gov/study/NCT05067660 (2021). Accessed 31 Mar 2024.

15. ClinicalTrials.gov: Androgen deprivation therapy (ADT) versus stereotactic body radiotherapy (SBRT) for oligometastatic prostate cancer: a prospective randomized control clinical trial (NCT04599686). https://clinicaltrials.gov/show/NCT04599686 (2020). Accessed 22 Mar 2024.

16. Zhao X, Wang T, Ye Y, Li J, Gao X, Zhang H. Stereotactic body radiotherapy (SBRT) versus androgen deprivation therapy (ADT) for oligometastatic prostate cancer: protocol for a prospective randomised control clinical trial. BMJ Open. 2022;12:e051371.

17. ClinicalTrials.gov: Phase 2 randomized total eradication of metastatic lesions following definitive radiation to the prostate in de novo oligometastatic prostate cancer trial (TERPS) (NCT05223803). https://clinicaltrials.gov/study/NCT05223803 (2022). Accessed 29 Mar 2024.

18. International Clinical Trials Registry Platform: Prostate only radiotherapy versus radiotherapy to all sites in oligo-metastatic prostate cancer - a phase II randomized open label study (PROS-O) (CTRI/2022/10/046239). https://trialsearch.who.int/Trial2.aspx?TrialID=CTRI/2022/10/046239 (2022). Accessed 23 Mar 2024.

19. ClinicalTrials.gov: Phase III study of stereotactic body radiation therapy (SBRT) plus standard of care in castration sensitive oligometastatic prostate cancer patients (START-MET) (NCT05209243). https://clinicaltrials.gov/study/NCT05209243 (2022). Accessed 29 Mar 2024.

20. ClinicalTrials.gov: Apalutamide and stereotactic body radiation therapy for low burden metastatic hormone sensitive prostate cancer, a randomized trial - PERSIAN (NCT05717660). https://clinicaltrials.gov/study/NCT05717660 (2023). Accessed 22 Mar 2024.

21. Francolini G, Porreca A, Facchini G, Santini D, Bruni A, Simoni N, et al. PERSIAN trial (NCT05717660): an ongoing randomized trial testing androgen deprivation therapy, apalutamide and stereotactic body radiotherapy. An alternative "triplet" for oligometastatic hormone sensitive prostate cancer patients. Med Oncol. 2023;41:39.

22. ClinicalTrials.gov: A randomized phase III trial of stereotactic ablative radiotherapy for patients with up to 10 oligometastases and a synchronous primary tumor (SABR-SYNC) (NCT05717166). https://clinicaltrials.gov/study/NCT05717166 (2023). Accessed 31 Mar 2024.

23. ClinicalTrials.gov: Studying treatments in patients receiving androgen deprivation therapy (ADT) and androgen receptor signalling inhibitors (ARSI) for metastatic prostate cancer: evaluation of drug and radiation efficacy: a 2nd multi-arm multi-stage randomised controlled trial (STAMPEDE2) (NCT06320067). https://clinicaltrials.gov/study/NCT06320067 (2024). Accessed 23 Mar 2024.
