# Supplementary material for: Stereotactic body radiotherapy as metastasis-directed therapy in oligometastatic prostate cancer: a systematic review and meta-analysis of randomized controlled trials
Source: Radiat Oncol. 2024 Dec 17;19:173. doi: 10.1186/s13014-024-02559-7 (PMC11654405; doi:10.1186/s13014-024-02559-7)
Supplement: Supplementary file 5 — Additional file 5. [file 13014_2024_2559_MOESM5_ESM.doc]

**Additional file 5.** Detailed characteristics of included studies

**STOMP [1-4]**

| ***Study characteristics*** | | |
| --- | --- | --- |
| Methods | **Registration number(s):** NCT01558427  **Study design:** RCT with two parallel arms. Phase II.  **Unit of allocation:** Participant  **Setting:** Multicenter. 6 centers.  **Country of the study coordinator:** Belgium^P^  **Enrollment country/countries:** Belgium  **Enrollment period:** August 1, 2012–August 31, 2015  **Screening procedures:** NI  **Number screened for inclusion:** 208  **Number randomized:** 62  **Randomization procedures:** Blocks with variable sizes of two and four, stratified by PSA-DT ≤3 *vs* >3 months and nodal *vs* non-nodal metastases  **Randomization ratio:** 1:1  **Other treatment arms:** None  **Follow-up period:** NI  **Follow-up time:** NI (for the whole study population median 5.3 years [IQR 4.3–6.3])  **Funding source(s):** Kom op tegen Kanker, Belgium | |
| Participants | 56 participants (25 received SBRT in IG, 31 in CG; 6 received surgery alone in IG as decided by the multidisciplinary team)  **Inclusion criteria:**   - ≥18 years;^P^ WHO PS 0–1 - Previous treatment with curative intent (radical prostatectomy, RT, or both) for pathologically confirmed PCa - 1–3 extracranial metastases (N1 or M1) on choline PET-CT; controlled primary tumor, i.e., on mpMRI or negative prostate (bed) biopsy - PSA recurrence as defined in EAU–ESTRO–SIOG guidelines [5] - Willing to give signed informed consent   **Exclusion criteria:**   - Symptomatic metastases - PSA recurrence during systemic treatment with LHRH agonist/antagonist, anti-androgen, or estrogen; previous MDT; previous cytotoxic treatment for PCa - Treatment with medication known to affect PSA levels during the prior month; testosterone <173 nmol/­L^a^   **Staging imaging modality/modalities:** Choline PET-CT  **Age (years):** NI  **Controlled primary tumor:** Yes  **Castration-resistant disease:** No  **Sites and numbers of treated metastases:** Node, node and bone, or bone. NI for IG *vs* 1 metastasis 29.0%, 2 metastases 32.3%, 3 metastases 38.7% in CG.  **PSA (µg/L):** NI *vs* median 3.8^b^ (IQR 0.8–9.6)  **PSA-DT:** NI *vs* ≤3 months 32.3%, >3 months 67.7%  **Previous use and type of systemic treatment:** ADT allowed at PCa diagnosis. NI *vs* ADT 48.4%.  **Previous conventional nodal RT:** Pelvic RT allowed  **Previous SBRT:** No  **Previous use and type(s) of alternative MDT:** No  **State(s) of OMD according to ESTRO–EORTC consensus recommendation:** Metachronous oligorecurrence  **Disease risk category at primary radical prostatectomy or RT:** NI  **Latest treatment with curative intent to prostate:** NI *vs* radical prostatectomy 16.1%, RT 25.8%, radical prostatectomy and RT 58.1% at PCa diagnosis  **Disease-free interval**^c^**:** NI | |
| Interventions | intervention group  SBRT, total dose 30 Gy (10 Gy × 3 fractions). >48 hours and <96 hours between fractions. Total treatment length >4 and <8 days^d^). NI on timing. All participants received allocated intervention.  control group  Surveillance | |
| Outcomes | critical outcomes (definition, follow-up)  **Overall survival:** Overall survival (Time to death,^P^ NI)  **Incidence proportion of grade ≥3 toxicity at 5 years or longest follow-up:** Toxicity grade ≥3 (CTCAE v4.0, NI)  additional important outcomes  **PFS:** PFS (Time to PSA/clinical/radiographic progression,^e^ start of ADT, death, or study withdrawal, NI)  **Local control at 5 years or longest follow-up:** Symptomatic or local progression (see footnote,^f^ NI)  **Incidence proportion of grade 5 toxicity at 5 years or longest follow-up:** Toxicity grade 5 (CTCAE v4.0, NI)  **HRQoL at 3 months:** Quality of life (EORTC QLQ-C30, QLQ-PR25, 3 months)  **HRQoL at 5 years or longest follow-up:** Quality of life (EORTC QLQ-C30, QLQ-PR25, 1 year)  **Systemic therapy-free survival:** ADT-free survival (Time to start of palliative ADT or death, NI)  other outcomes  **Local PFS:** NP  **Distant PFS:** Radiographic PFS (Time to new nodal, bone, or visceral metastasis or death, NI)  **Follow-up procedures:** Clinical examination, toxicity assessment, and PSA testing every 3 months until ADT-free survival end point was met, after per the EAU–ESTRO–SIOG guidelines [5]. Choline PET-CT performed at PSA or symptomatic progression. Patient-reported HRQoL at 3 months and annually. | |
| ***Risk of bias*** | | |
| **Bias** | **Authors’ judgement** | **Support for judgement** |
| Randomization process | Low risk |  |
| Deviations from intended interventions | High risk | 5 of 31 participants (16%) in the CG crossed over to the IG |
| Missing outcome data | Low risk | No separate outcome data for participants in the IG treated with SBRT for overall survival, PFS, HRQoL, systemic therapy-free survival, and distant PFS but there was no evidence of bias due to missing outcome data |
| Measurement of the outcome | Some concerns  **Overall survival:** Low  **Incidence proportion of grade ≥3 toxicity at 5 years or longest follow-up:** Some concerns  **PFS:** Some concerns  **Local control at 5 years or longest follow-up:** Some concerns  **Incidence proportion of grade 5 toxicity at 5 years or longest follow-up:** Some concerns  **HRQoL at 3 months:** Some concerns  **HRQoL at 5 years or longest follow-up:** Some concerns  **Systemic therapy-free survival:** Some concerns  **Distant PFS:** Some concerns | Assessors were aware of intervention received and could have influenced assessments except for overall survival, but it was considered unlikely |
| Selection of the reported result | Low risk |  |
| Overall judgement | High risk |  |

**SABR-COMET [6-9]**

| ***Study characteristics*** | | |
| --- | --- | --- |
| Methods | **Registration number(s):** NCT01446744  **Study design:** RCT with two parallel arms. Phase II.  **Unit of allocation:** Participant  **Setting:** Multicenter. 10 centers.  **Country of the study coordinator:** NI  **Enrollment country/countries:** Canada, the Netherlands, UK, Australia  **Enrollment period:** February 10, 2012–August 30, 2016  **Screening procedures:** NI  **Number screened for inclusion:** Not assessed  **Number randomized:** 99  **Randomization procedures:** Computer-generated randomization with permuted blocks of nine, stratified by number of metastases (1–3 *vs* 4–5)  **Randomization ratio:** 2:1  **Other treatment arms:** None  **Follow-up period:** NI  **Follow-up time:** NI (for the whole study population median 5.7 years [95% CI 5.1–7.0])  **Funding source(s):** The Ontario Institute for Cancer Research, London Regional Cancer Program Catalyst Grant | |
| Participants | 16 participants (14 in IG and 2 in CG; 83 with other primary tumor types included in the study)  **Inclusion criteria:**   - ≥18 years; ECOG PS 0–1; life expectancy ≥6 months - Histologically verified malignancy^P^ - 1–5 metastases with ≤3 metastases per organ on PET-CT or CT and bone scan ± MRI spine if vertebral metastases - Definitively treated primary tumor ≥3 months prior, without radiographic evidence of progression - Possible to treat all metastases with SABR - Consensus during multidisciplinary tumor board meeting or quality-assurance rounds that study entry was suitable, e.g., surgery not an alternative for treating all metastases^P^ - No chemotherapy or targeted therapy 4 weeks before or during SABR - If previously treated metastases:   - Surgery/radiofrequency ablation/RT to a metastasis controlled on imaging: patients were eligible, metastasis not treated with SABR   - Surgery to a metastasis not controlled in imaging: patients were eligible, metastasis treated with SABR   - Radiofrequency ablation or RT: patients were ineligible^P^ - Willing to give informed consent^P^   **Exclusion criteria:**   - Significant medical comorbidities prohibiting RT - Femoral bone metastasis; 1–3 brain metastases without other dissemination; dominant brain metastasis needing surgical decompression - Not possible to treat all sites of active disease;^P^ previous RT to a site needing treatment - Signs of spinal cord compression (clinical or radiographic);^P^ tumor within 3 mm of spinal cord on MRI - Complete response to first line of chemotherapy;^P^ malignant pleural effusion   **Staging imaging modality/modalities:** CT or MRI of the brain for primary tumors with tendency for brain metastasis (prostate cancer denoted as having low propensity^P^). CT neck/chest/abdomen/pelvis and bone scan or PET-CT. Spine MRI if vertebral metastases.  **Age (years):** NI  **Controlled primary tumor:** Yes  **Castration-resistant disease:** Allowed^P^  **Sites and numbers of treated metastases:** Potential sites: Node, bone, lung, liver, brain, adrenal, and pararenal (CG only). NI on number of metastases.  **PSA (µg/L):** NI  **PSA-DT (months):** NI  **Previous use and type of systemic treatment:** Previous^P^ or ongoing chemotherapy or hormonal therapy^P^ allowed.  **Previous conventional nodal RT:** Allowed^P^  **Previous SBRT:** Allowed^P^  **Previous use and type(s) of alternative MDT:** Allowed^P^  **State(s) of OMD according to ESTRO–EORTC consensus recommendation:** All states allowed^P^  **Disease risk category at primary radical prostatectomy or RT:** NI  **Latest treatment with curative intent to prostate:** NI  **Disease-free interval**^c^**:** NI | |
| Interventions | intervention group  SABR, total dose 16–60 Gy (5–24 Gy × 1–12 fractions). Single dose, daily, or every 2^nd^ day. Total treatment length 1 day–3.5 weeks,^d,P^ to all (uncontrolled) metastases. NI on timing. 64 of 66 allocated to intervention arm received intervention (other 2 had >5 metastases at baseline). SOC.  control group  SOC | |
| Outcomes | critical outcomes (definition, follow-up)  **Overall survival:** Overall survival (Time to death, NI)  **Incidence proportion of grade ≥3 toxicity at 5 years or longest follow-up:** Toxicity grade ≥3 (CTCAE v4, NI)  additional important outcomes  **PFS**: PFS (Time to progression according to RECIST 1.0^P^ or death, NI)  **Local control at 5 years or longest follow-up:** Lesional control rate (RECIST 1.0,^P^ NI)  **Incidence proportion of grade 5 toxicity at 5 years or longest follow-up:** Toxicity grade 5 (CTCAE v4, NI)  **HRQoL at 3 months:** Quality of life (FACT-G, 6 months)  **HRQoL at 5 years or longest follow-up:** Quality of life (FACT-G, 6 years)  **Systemic therapy-free survival:** NP  other outcomes  **Local PFS:** NP  **Distant PFS:** Time to development of new metastases (NI)  **Follow-up procedures:** H&P and QoL assessment every 3 months year 1–2, every 6 months year 3–5 and annually year 6–10. Toxicity assessment every 3 months year 1–2, at 3, 6, 12, 18, and 24 months year 1–2, every 6 months year 3–5, and annually year 6–10. CT chest/abdomen/pelvis and bone scan at 3, 6, 12, 18, and 24 months and every 6 months year 3–5.^P^ | |
| ***Risk of bias*** | | |
| **Bias** | **Authors’ judgement** | **Support for judgement** |
| Randomization process | Low risk |  |
| Deviations from intended interventions | Low risk |  |
| Missing outcome data | Low risk | No separate outcome data for participants with PCa, but there was no evidence of bias due to missing outcome data |
| Measurement of the outcome | Some concerns  **Overall survival:** Low risk  **Incidence proportion of grade ≥3 toxicity at 5 years or longest follow-up:** Some concerns  **PFS:** Some concerns  **Local control at 5 years or longest follow-up:** Some concerns  **Incidence proportion of grade 5 toxicity at 5 years or longest follow-up:** Some concerns  **HRQoL at 3 months:** Some concerns  **HRQoL at 5 years or longest follow-up:** Some concerns  **Distant PFS:** Some concerns | Assessors were aware of intervention received and could have influenced assessments except for overall survival, but it was considered unlikely |
| Selection of the reported result | Low risk |  |
| Overall judgement | Some concerns |  |

**ORIOLE [3, 10, 11]**

| ***Study characteristics*** | | |
| --- | --- | --- |
| Methods | **Registration number(s):** NCT02680587  **Study design:** RCT with two parallel arms. Phase II.  **Unit of allocation:** Participant  **Setting:** Multicenter. 3 centers.  **Country of the study coordinator:** USA  **Enrollment country/countries:** USA  **Enrollment period:** May 25, 2016–March 5, 2018  **Screening procedures:** NI  **Number screened for inclusion:** 80  **Number randomized:** 54  **Randomization procedures:** Interactive web response system. Minimization based on stratification by initial treatment (surgery *vs* RT), previous ADT (yes *vs* no), and PSA-DT (<6 months *vs* 6–14.9 months).  **Randomization ratio:** 2:1  **Other treatment arms:** None  **Follow-up period:** May 25, 2016–May 20, 2019  **Follow-up time:** Median 18.8 months (range 5.8–35.0)  **Funding source(s):** The Nesbitt-McMaster Foundation, Ronald Rose and Joan Lazar, the Movember Foundation, the Prostate Cancer Foundation, the National Cancer Institute, SDW/DT and Shanahan Cancer Research Funds, the US National Institutes of Health Director’s New Innovator Award, the Virginia and D.K. Ludwig Fund for Cancer Research, the CRK Faculty Scholar Fund and the Transdisciplinary Integration of Population Science Program of Sidney Kimmel Cancer Center–Jefferson Health | |
| Participants | 54 participants (36 in IG and 18 in CG)  **Inclusion criteria:**   - ≥18 years; ECOG PS ≤2 - Pathologically confirmed PCa; previous definitive surgery or RT of the primary tumor; ADT or other systemic therapy allowed during initial or salvage treatment - 1–3 asymptomatic metastases developing within 6 months on CT, MRI and/or bone scan; ≤5.0 cm or ≤250 cm^2^ - PSA ≥0.5 and ≤50 µg/L^b^; testosterone ≥4.3 nmol/L^g^; PSA-DT <15 months; leukocytes >2 000/μL; ANC >1 000/μL; platelets >50 000/μL - Able to understand and give informed consent   **Exclusion criteria:**   - >3 years of ADT; ADT or other systemic therapy during prior 6 months; castration-resistant disease - PSMA PET-CT or PET-MRI within 6 months with positive lesions not visible on conventional imaging; suspected pulmonary and/or liver metastases >10 mm in longest diameter - Spinal cord compression or imminent spinal cord compression - Inability to lie flat or tolerate PET-CT, MRI, or SABR - Creatinine >3x ULN; total bilirubin >3x ULN; liver transaminases >5x ULN - Receiving other investigational agents or partaking in concurrent treatment protocols - Not willing to sign informed consent document   **Staging imaging modality/modalities:** CT, MRI, and/or bone scan  **Age (years):** Median 68 (range 61–70) in IG *vs* 68 (64–76) in CG  **Controlled primary tumor:** NI  **Castration-resistant disease:** No  **Sites and numbers of treated metastases:** Node only: 58%, node and bone/bone: 42%, mean 2.03 metastases/participant *vs* node only: 67%, node and bone/bone: 33%, mean 1.66 metastases/participant  **PSA (µg/L):** Median 6^b,h^ (range 2–13) *vs* 7 (3–17)  **PSA-DT (months):** Median 8 (range 4–11) *vs* 6 (4–11)  **Previous use and type of systemic treatment:** ADT or other systemic therapy allowed during initial or salvage treatment. ADT 42% *vs* 28%.  **Previous conventional nodal RT:** Salvage RT to pelvis allowed  **Previous SBRT:** NI  **Previous use and type(s) of alternative MDT:** NI  **State(s) of OMD according to ESTRO–EORTC consensus recommendation:** Metachronous oligorecurrence  **Disease risk category at primary radical prostatectomy or RT:** NI  **Latest treatment with curative intent to prostate:** Previous definitive surgery 83%, RT 17% in both groups. Salvage RT to the prostate bed allowed.  **Disease-free interval**^c^**:** NI | |
| Interventions | intervention group  SABR, total dose 19.5–48.0 Gy (5–12 Gy × 3–5). NI on time between fractions or total treatment length. Start within 3 weeks of simulation. All participants received allocated intervention.  control group  Observation (Allowed to cross over at progression or 6 months) | |
| Outcomes | critical outcomes (definition, follow-up)  **Overall survival:** Overall survival (Time to death,^P^ 6 months until crossover)  **Incidence proportion of grade ≥3 toxicity at 5 years or longest follow-up:** Adverse effects grade ≥3 (CTCAE v4.0, 6 months until crossover)  additional important outcomes  **PFS:** PFS (Time to PSA/clinical/radiographic progression,^e^ start of ADT, death, or study withdrawal, 6 months until crossover)  **Local control at 5 years or longest follow-up:** NP  **Incidence proportion of grade 5 toxicity at 5 years or longest follow-up:** Adverse effects grade 5 (CTCAE v4.0, 6 months until crossover)  **HRQoL at 3 months:** Quality of life (BPI-SF, 3, 6 months^P^)  **HRQoL at 5 years or longest follow-up:** NP  **Systemic therapy-free survival:** NP  other outcomes  **Local PFS:** NP  **Distant PFS:** Radiographic PFS (Time to new nodal, bone, or visceral metastasis or death, 6 months until crossover)  **Follow-up procedures:** H&P, PSA testing, toxicity,^P^ and QoL^P^ evaluation at 3 and 6 months. CT chest/abdomen/pelvis and bone scan and PSMA PET-CT (IG only) at 6 months. Routine follow-up every 3 months. | |
| ***Risk of bias*** | | |
| **Bias** | **Authors’ judgement** | **Support for judgement** |
| Randomization process | Low risk |  |
| Deviations from intended interventions | Low risk | 15 of 18 participants in the CG crossed over to the IG at progression or 6 months as permitted; only follow-up until 6 months included in this review |
| Missing outcome data | Low risk | No reported HRQoL data, only that there was no difference, but there was no evidence of bias due to missing outcome data |
| Measurement of the outcome | Some concerns  **Overall survival:** Low  **Incidence proportion of grade ≥3 toxicity at 5 years or longest follow-up:** Some concerns  **PFS:** Some concerns  **Incidence proportion of grade 5 toxicity at 5 years or longest follow-up:** Some concerns  **HRQoL at 3 months:** Some concerns  **Distant PFS:** Some concerns | Assessors were aware of intervention received and could have influenced assessments except for overall survival, but it was considered unlikely |
| Selection of the reported result | Low risk |  |
| Overall judgement | Some concerns |  |

**ARTO [12]**

| ***Study characteristics*** | | |
| --- | --- | --- |
| Methods | **Registration number(s):** NCT03449719  **Study design:** RCT with two parallel arms. Phase II.  **Unit of allocation:** Participant  **Setting:** Multicenter. 16 centers.  **Country of the study coordinator:** Italy^i^  **Enrollment country/countries:** Italy  **Enrollment period:** January 2019–September 2022  **Screening procedures:** All patients with castration-resistant PCa in the outpatient clinic were screened for inclusion^i^  **Number screened for inclusion:** NI  **Number randomized:** 157  **Randomization procedures:** Computer-generated random number table, stratification by random permuted blocks according to center, performance status (0–1 *vs* >1), and number of metastases (1 *vs* >1)  **Randomization ratio:** 1:1  **Other treatment arms:** No  **Follow-up period:** January 2019–March 2023^i^  **Follow-up time:** Median 24.9 months (IQR 17.1–35.8)  **Funding source(s):** Janssen-Cilag SpA | |
| Participants | 157 participants (75 in IG and 82 in CG)  **Inclusion criteria:**   - ≥18 years^P^ - Metastatic castration-resistant PCa - 1–3 nodal or bone metastases on CT and/or bone scan or choline, fluciclovine, or PSMA PET-CT - No or mild symptoms^P^ - Receipt of abiraterone acetate for 30±3 days before potentially starting SBRT (in the intervention arm)^P^ - Willing to participate and having signed informed consent document^P^   **Exclusion criteria:**   - Previous treatment for metastatic castration-resistant PCa (except hormonal therapy^P^) - >3 metastases^P^ - Visceral metastases - Contraindications or hypersensitivity to abiraterone acetate, GnRH agonist/antagonist, or RT^P^ - Any condition were, according to the investigator, participation would not be in the best interest of the patient^P^   **Staging imaging modality/modalities:** CT and/or bone scan or choline, fluciclovine, or PSMA PET-CT  **Age (years):** Median 74 (IQR 68–79) in both IG and CG  **Controlled primary tumor:** 14 participants (9%) had uncontrolled primary tumors^i^  **Castration-resistant disease:** Yes  **Sites and numbers of treated metastases:** Node: 44% in IG *vs* 53.6% in CG, bone: 56% *vs* 46.4%  **PSA (µg/L):** Median 3.42^b,j^ (IQR 1.47–9.36) *vs* 3.42 (1.46–9.35)  **PSA-DT (months):** NI  **Previous use and type of systemic treatment:** Hormonal therapy. Previous systemic therapy for castration-resistant disease not allowed.  **Previous conventional nodal RT:** Allowed^i^  **Previous SBRT:** Allowed^i^  **Previous use and type(s) of alternative MDT:** Allowed^i^  **State(s) of OMD according to ESTRO–EORTC consensus recommendation:** All allowed with castration-resistant disease  **Disease risk category at primary radical prostatectomy or RT:** Intermediate: 10 participants (6%), high: 147 participants (94%)^i^  **Latest treatment with curative intent to prostate:** Radical prostatectomy: 47 participants (30%), definitive RT: 32 participants (20%), postoperative RT: 41 participants (26%)^i^  **Disease-free interval**^c^**:** NI | |
| Interventions | intervention group  SBRT, total dose 16–40 Gy (6.5–16 Gy × 1–5 fractions). 0–1 day between fractions.^i^ Total treatment length 3–18 days.^i^ Start of SBRT 30±3 days after randomization.^i^ Abiraterone acetate + prednisone, ADT. 74 of 75 participants received allocated interventions.  control group  Abiraterone acetate + prednisone, ADT. | |
| Outcomes | critical outcomes (definition, follow-up)  **Overall survival:** Overall survival (Time to death, median 24.9 months [IQR 17.1–35.8])  **Incidence proportion of grade ≥3 toxicity at 5 years or longest follow-up:** Adverse events grade ≥3 (CTCAE v4.03, median 24.9 months [IQR 17.1–35.8])  additional important outcomes  **PFS:** PFS (Time to PSA/radiographic progression,^k^ start of following treatment, or death, median 24.9 months [IQR 17.1–35.8])  **Local control at 5 years or longest follow-up:** NP  **Incidence proportion of grade 5 toxicity at 5 years or longest follow-up:** Adverse events grade 5 (CTCAE v4.03, median 24.9 months [IQR 17.1–35.8])  **HRQoL at 3 months:** Quality of life (EORTC QLQ-C30, 3 months)  **HRQoL at 5 years or longest follow-up:** Quality of life (EORTC QLQ-C30, BPI-SF, 1 year^P^)  **Systemic therapy-free survival:** NA  other outcomes  **Local PFS:** NP  **Distant PFS:** NP  **Follow-up procedures:** Toxicity assessments at every study visit and in case of treatment discontinuation. PSA testing and QoL assessments^P^ every 3 months. Radiological assessments (with the same modalities as baseline) at clinical or PSA progression per the Prostate Cancer Working Group 3 criteria [13]. | |
| ***Risk of bias*** | | |
| **Bias** | **Authors’ judgement** | **Support for judgement** |
| Randomization process | Low risk |  |
| Deviations from intended interventions | Low risk | One participant in the IG did not want SBRT, but it was unlikely to have affected outcomes |
| Missing outcome data | High risk  **Overall survival:** Low risk  **Incidence proportion of grade ≥3 toxicity at 5 years or longest follow-up:** Low risk  **PFS:** Low risk  **Incidence proportion of grade 5 toxicity at 5 years or longest follow-up:** Low risk  **HRQoL at 3 months:** High risk | HRQoL at 3 months was reported in only a subset (18%) of the participants |
| Measurement of the outcome | Some concerns  **Overall survival:** Low risk  **Incidence proportion of grade ≥3 toxicity at 5 years or longest follow-up:** Some concerns  **PFS:** Some concerns  **Incidence proportion of grade 5 toxicity at 5 years or longest follow-up:** Some concerns  **HRQoL at 3 months:** Some concerns | Assessors were aware of intervention received and could have influenced assessments except for overall survival, but it was considered unlikely |
| Selection of the reported result | Low risk |  |
| Overall judgement | High risk |  |

**EXTEND [14]**

| ***Study characteristics*** | | |
| --- | --- | --- |
| Methods | **Registration number(s):** NCT03599765  **Study design:** Basket RCT with two parallel arms. Phase II.  **Unit of allocation:** Participant  **Setting:** Multicenter. 3 centers.  **Country of the study coordinator:** USA^P^  **Enrollment country/countries:** USA  **Enrollment period:** September 2018–November 2020  **Screening procedures:** NI  **Number screened for inclusion:** 124  **Number randomized:** 87  **Randomization procedures:** Dynamic randomization method stratified by number of metastases (1–2 *vs* 3–5), number of previous systemic therapies  (0–1 *vs* >1), use of second-generation androgen receptor inhibitor (yes *vs* no), and length of hormone therapy (<12 *vs* ≥12 weeks)  **Randomization ratio:** 1:1  **Other treatment arms:** No  **Follow-up period:** September 2018–January 2022  **Follow-up time:** Median 22.0 months (range 11.6–39.2)  **Funding source(s):** Cancer Prevention and Research Institute of Texas, National Cancer Institute (National Institutes of Health) | |
| Participants | 87 participants (43 in IG and 44 in CG)  **Inclusion criteria:**   - ≥18 years; ECOG PS ≤2 - Pathologically confirmed PCa - 1–5 metastases possible to treat with MDT - Use of hormone therapy (LHRH agonist/antagonist ± second-generation androgen receptor inhibitor) ≥2 months - Previously received ≤4 lines of systemic therapy for metastatic disease^P^ - ANC ≥500/µL; platelets ≥25 000/µL; Hb ≥7 g/dL; total bilirubin ≤25.7 µmol/L^l^ (for patients with Gilbert’s syndrome <51.3 µmol/L) or direct bilirubin ≤ULN if total bilirubin >25.7 µmol/L; AST and ALT ≤3x ULN or ≤5x ULN in case of liver metastases^P^   **Exclusion criteria:^P^**   - Rheumatic disease that hinders safe delivery of RT - Psychiatric or substance abuse disorder interfering with participation - Another primary cancer that carries a significant risk to the patient’s life according to the treating physician and investigational team - Metastatic effusion (e.g., pleural effusion) - Diffuse metastatic disease (e.g., leptomeningeal disease) that is not possible to treat definitively   **Staging imaging modality/modalities:** CT and bone scan (IG 77% *vs* CG 75%) or fluciclovine PET-CT (23% *vs* 25%)  **Age (years):** Median 67 (IQR 63–72) in both IG and CG  **Controlled primary tumor:** Previous definitive treatment of the primary tumor: 72%  **Castration-resistant disease:** 9% *vs* 7%  **Sites and numbers of treated metastases:** Regional lymph nodes: 7% *vs* 7%, distant lymph nodes: 26% *vs* 25%, bone ± lymph nodes: 65% *vs* 66%, other sites ± bone, lymph nodes: 2% *vs* 2%. 1 metastasis: 28% *vs* 48%, 2 metastases: 42% *vs* 30%, 3: 19% *vs* 9%, 4–5: 12% *vs* 14%.  **PSA (µg/L):** ≤0.2^b^: 53% *vs* 61%, >0.2–<2.0: 35% *vs* 32%, ≥2: 12% *vs* 7%  **PSA-DT (months):** NI  **Previous use and type of systemic treatment:** No lines: 72% *vs* 70%, 1 line: 23% *vs* 27%, 2 lines: 5% *vs* 2%  **Previous conventional nodal RT:** NI  **Previous SBRT:** NI  **Previous use and type(s) of alternative MDT:** NI  **State(s) of OMD according to ESTRO–EORTC consensus recommendation:** All states allowed  **Disease risk category at primary radical prostatectomy or RT:** NI  **Latest treatment with curative intent to prostate:** NI  **Disease-free interval**^c^**:** NI | |
| Interventions | intervention group  RT as MDT, recommended total doses 12–70 Gy (2.3–27 Gy × 1–28).^m,P^ NI on time between fractions or length of treatment. Recommended to be completed within 4 weeks of enrollment.^P^ Intermittent hormone therapy (LHRH agonist/antagonist ± second-generation androgen receptor inhibitor ≥6 months, with planned break 4–8 months after enrollment). Prostate RT if previously untreated primary tumor. All participants received allocated intervention.  control group  Intermittent hormone therapy. Prostate RT if previously untreated primary tumor. | |
| Outcomes | critical outcomes (definition, follow-up)  **Overall survival:** Overall survival (Time to death,^P^ median 22.0 months [range 11.6–39.2])  **Incidence proportion of grade ≥3 toxicity at 5 years or longest follow-up:** Adverse events grade ≥3 (CTCAE v4.0, median 22.0 months [range 11.6–39.2])  additional important outcomes  **PFS:** PFS (Time to PSA/clinical/radiographic progression^n^ or death, median 22.0 months [range 11.6–39.2])  **Local control at 5 years or longest follow-up:** NP  **Incidence proportion of grade 5 toxicity at 5 years or longest follow-up:** Adverse events grade 5 (CTCAE v4.0, median 22.0 months [range 11.6–39.2])  **HRQoL at 3 months:** Quality of life (CES-D, MDASI, SF-12, 3 months^P^)  **HRQoL at 5 years or longest follow-up:** Quality of life (CES-D, MDASI, SF-12, median 22.0 months [range 11.6–39.2])  **Systemic therapy-free survival:** NA  other outcomes  **Local PFS:** NP  **Distant PFS:** Time to new lesion failure (Time to new lesion outside of the initial lesions present,^P^ 2 years)  **Follow-up procedures:** PSA measurement, physical examination,^P^ and toxicity assessment^P^ every 3 months year 1–2, thereafter every 4 months. QoL questionnaire optional.^P^ Radiographic evaluation after PSA increase of ≥1 µg/L^b^ above nadir or other criteria for disease progression were met (clinical^P^). | |
| ***Risk of bias*** | | |
| **Bias** | **Authors’ judgement** | **Support for judgement** |
| Randomization process | Low risk |  |
| Deviations from intended interventions | Low risk |  |
| Missing outcome data | High risk  **Overall survival:** Low risk  **Incidence proportion of grade ≥3 toxicity at 5 years or longest follow-up:** Low risk  **PFS:** Low risk  **Incidence proportion of grade 5 toxicity at 5 years or longest follow-up:** Low risk  **HRQoL at 3 months:** High risk  **HRQoL at 5 years or longest follow-up:** High risk  **Distant PFS:** Low risk | Baseline HRQoL assessment only available for a subset (43%) of participants |
| Measurement of the outcome | High risk  **Overall survival:** Low risk  **Incidence proportion of grade ≥3 toxicity at 5 years or longest follow-up:** Some concern  **PFS:** Some concern  **Incidence proportion of grade 5 toxicity at 5 years or longest follow-up:** Some concern  **HRQoL at 3 months:** High risk  **HRQoL at 5 years or longest follow-up:** High risk  **Distant PFS:** Some concern | Assessors were aware of intervention received and could have influenced assessments except for overall survival, but it was considered unlikely  HRQoL reporting was optional for participants, and willingness to respond may have been unbalanced between arms |
| Selection of the reported result | Low risk |  |
| Overall judgement | High risk |  |

**CORE [15, 16]**

| ***Study characteristics*** | | |
| --- | --- | --- |
| Methods | **Registration number(s):** NCT02759783. ISRCTN45961438.^P^  **Study design:** RCT with two parallel arms. Phase II.  **Unit of allocation:** Participant  **Setting:** Multicenter. 30 centers.  **Country of the study coordinator:** UK^P^  **Enrollment country/countries:** UK, Australia  **Enrollment period:** November 2016–February 2019  **Screening procedures:** Logging of all potentially eligible patients with 1–3 metastases at each trial site^P^  **Number screened for inclusion:** NI  **Number randomized:** 245  **Randomization procedures:** Minimization with a random component^P^  **Randomization ratio:** 1:1  **Other treatment arms:** No  **Follow-up period:** NI  **Follow-up time:** NI (for the whole study population median 42.5 months [IQR 35.9–48.8])  **Funding source(s):** Cancer Research UK^P^ | |
| Participants | 180 participants (65 with other primary tumor types included in the study)  **Inclusion criteria:**   - ≥18 years; WHO PS ≤2; life expectancy >6 months^P^ - Pathologically confirmed PCa^P^ - Metachronous metastatic disease; controlled primary tumor^P^ - 1–3 metastases in 1–2 organ systems^P^ - No previous, or <8 weeks of, systemic treatment for metastatic disease^P^ - Metastases defined on imaging and suitable for SBRT^P^ - Controlled metastases if previously treated with local ablative therapy^P^ - Acceptable organ function that allows SBRT^P^ - Written informed consent^P^   **Exclusion criteria:^P^**   - Intracranial metastases - Malignant pleural effusion and/or peritoneal disease - Locoregional nodal recurrence where surgery is SOC and a treatment option - Metastasis of >6 cm (>5 cm if lung metastasis) - Previous or ongoing treatment with abiraterone, enzalutamide, or chemotherapy - Recurrence during ADT, initiated without initial staging, or combined androgen blockade, initiated for biochemical recurrence only - Other cancer (excluding basal cell carcinoma or squamous cell carcinoma of the skin) during the prior 2 years or deemed to considerably increase the risk of death during the upcoming 5 years - Spinal cord compression or indication for spine RT within 24 hours - Not possible to safely deliver SBRT to a site due to previous RT - Health conditions that hinder imaging for staging or follow-up, needed preparations for SBRT, or safe delivery of SBRT - Enrollment would postpone management in a way that cannot be permitted - Personal circumstances hindering adherence to trial procedures and follow-up   **Staging imaging modality/modalities:** CT and bone scan or choline/PSMA PET-CT or WBMRI^P^  **Age (years):** NI  **Controlled primary tumor:** Yes^P^  **Castration-resistant disease:** Allowed^P^  **Sites and numbers of treated metastases:** NI  **PSA (µg/L):** NI  **PSA-DT (months):** NI  **Previous use and type of systemic treatment:** Allowed in adjuvant setting only^P^  **Previous conventional nodal RT:** Allowed^P^  **Previous SBRT:** Allowed^P^  **Previous use and type(s) of alternative MDT:** Allowed^P^  **State(s) of OMD according to ESTRO–EORTC consensus recommendation:** Metachronous oligorecurrence  **Disease risk category at primary radical prostatectomy or RT:** NI  **Latest treatment with curative intent to prostate:** NI  **Disease-free interval**^c^**:** ≥6 months^P^ | |
| Interventions | intervention group  SBRT, recommended total dose 24–60 Gy (7.5–18 Gy × 3–8). Daily or every 2^nd^ day. Total treatment length 7–21 days. Start within 6 weeks of randomization.^P^ NI on number of patients with PCa that received allocated intervention. SOC.  control group  SOC | |
| Outcomes | critical outcomes (definition, follow-up)  **Overall survival:** NP  **Incidence proportion of grade ≥3 toxicity at 5 years or longest follow-up:** NP  additional important outcomes  **PFS:** PFS (Time to radiographic ± PSA/clinical progression^o^ or death,^P^ NI)  **Local control at 5 years or longest follow-up:** NP  **Incidence proportion of grade 5 toxicity at 5 years or longest follow-up:** NP  **HRQoL at 3 months:** NP  **HRQoL at 5 years or longest follow-up:** NP  **Systemic therapy-free survival:** NP  other outcomes  **Local PFS:** NP  **Distant PFS:** NP  **Follow-up procedures:** Clinical examination, toxicity assessment, and PSA measurement every 3 months year 1–2 and every 6 months year 3–5. CT and bone scan, choline or PSMA PET-CT, or whole body MRI at 6, 12, and 24 months and after two rising PSA values from nadir measured at a ≥4 weeks interval. QoL assessments at 3, 6, 12, 18, and 24 months.^P^ | |
| ***Risk of bias*** | | |
| **Bias** | **Authors’ judgement** | **Support for judgement** |
| Randomization process | Low risk |  |
| Deviations from intended interventions | High risk | There were significant differences in completion of SOC between the IG and CG (62% *vs* 75%) |
| Missing outcome data | Low risk | No separate outcome data for participants with PCa, but there was no evidence of bias due to missing outcome data |
| Measurement of the outcome | Some concerns | Assessors were aware of intervention received and could have influenced assessment, but it was considered unlikely |
| Selection of the reported result | Low risk |  |
| Overall judgement | High risk |  |

**STOP [17-19]**

| ***Study characteristics*** | | |
| --- | --- | --- |
| Methods | **Registration number(s):** NCT02756793  **Study design:** RCT with two parallel arms. Phase II.  **Unit of allocation:** Participants  **Setting:** Multicenter. 8 centers.  **Country of the study coordinator:** NI  **Enrollment country/countries:** Canada  **Enrollment period:** February 2017–June 2021  **Screening procedures:** NI  **Number screened for inclusion:** NI  **Number randomized:** 90  **Randomization procedures:** Stratification by systemic therapy (cytotoxic *vs* non-cytotoxic)  **Randomization ratio:** 2:1  **Other treatment arms:** No  **Follow-up period:** NI  **Follow-up time:** NI (for the whole study population median 31 months)  **Funding source(s):** Lawson Health Research Institute^P^ | |
| Participants | 9 participants (7 in IG and 2 in CG; 81 with other primary tumor types included in the study)  **Inclusion criteria:**   - ≥18 years; ECOG PS 0–2; life expectancy >3 months^P^ - Metastatic disease detected on imaging^P^ - Receipt of systemic therapy during the prior 6 weeks^P^ for ≥3 months with previous response or stable disease - Oligoprogression of 1–5 metastases with ≤3 metastases per organ^P^ - Possible to safely treat all metastases with progression^P^ - No previous treatment with RT or radiofrequency ablation to the progressing metastases^P^ - Willing to give informed consent^P^   **Exclusion criteria:^P^**   - Significant medical comorbidity prohibiting RT - Previous treatment with RT to a site needing treatment - Malignant pleural effusion - Inability to treat all metastases with progression - Signs of spinal cord compression (clinical or radiographical); metastases within 3 mm of the spinal cord on MRI - Other condition making enrollment unsuitable per the judgement of the investigator   **Staging imaging modality/modalities:** NI  **Age (years):** NI  **Controlled primary tumor:** NI  **Castration-resistant disease:** 33% hormone-sensitive^i^  **Sites and numbers of treated metastases:** NI  **PSA (µg/L):** NI  **PSA-DT (months):** NI  **Previous use and type of systemic treatment:** Yes, during the prior 6 weeks for ≥3 months^P^  **Previous conventional nodal RT:** Allowed^P^  **Previous SBRT:** Allowed^P^  **Previous use and type(s) of alternative MDT:** Allowed^P^  **State(s) of OMD according to ESTRO–EORTC consensus recommendation:** Oligoprogression  **Disease risk category at primary radical prostatectomy or RT:** NI  **Latest treatment with curative intent to prostate:** NI  **Disease-free interval**^c^**:** NI  **Total numbers of metastases per participant (if oligoprogression):** NI | |
| Interventions | intervention group  SABR/SRT to all progressing metastases. Start as soon as possible after randomization.^P^ NI on doses, fractions, time between treatments, or length of intervention. NI on number of participants with PCa that received allocated intervention. SOC.  control group  SOC and SRT to brain metastases when indicated^P^ | |
| Outcomes | critical outcomes (definition, follow-up)  **Overall survival:** Overall survival (Time to death,^P^ NI)  **Incidence proportion of grade ≥3 toxicity at 5 years or longest follow-up:** Toxicity grade ≥3 related to RT^P^ (CTCAE v4 for each treated organ,^P^ NI)  additional important outcomes  **PFS:** PFS (Time to progression or death,^P^ NI)  **Local control at 5 years or longest follow-up:** Lesional control (NI)  **Incidence proportion of grade 5 toxicity at 5 years or longest follow-up:** Toxicity grade 5 related to RT^P^ (CTCAE v4 for each treated organ,^P^ NI)  **HRQoL at 3 months:** NP  **HRQoL at 5 years or longest follow-up:** Quality of life (FACT-G, 5 years^P^)  **Systemic therapy-free survival:** NP  other outcomes  **Local PFS:** NP  **Distant PFS:** NP  **Follow-up procedures:** NI | |
| ***Risk of bias*** | | |
| **Bias** | **Authors’ judgement** | **Support for judgement** |
| Randomization process | Some concern | There was no information on whether the allocation sequence was concealed until participants had been assigned to interventions |
| Deviations from intended interventions | High risk | 3 participants withdrew and 7 received high-dose or ablative therapies after assigned to the CG (in whole study population) |
| Missing outcome data | Low risk | No separate outcome data for participants with PCa, but there was no evidence of bias due to missing outcome data |
| Measurement of the outcome | Some concerns  **Overall survival:** Low risk  **Incidence proportion of grade ≥3 toxicity at 5 years or longest follow-up:** Some concerns  **PFS:** Some concerns  **LC:** Some concerns  **Incidence proportion of grade 5 toxicity at 5 years or longest follow-up:** Some concerns  **HRQoL at 5 years or longest follow-up:** Some concerns | Assessors were likely aware of intervention received, and there was not sufficient information to judge if it could have influenced the results except for overall survival. However, only an abstract and trial register entries were available at the time, and it was therefore not considered to warrant an assessment of high risk. |
| Selection of the reported result | Low risk |  |
| Overall judgement | High risk |  |

Reported in table as described by the study authors and denoted by superscript P if only reported in protocol. Eligibility criteria and interventions are described for the population relevant to this review.

Abbreviations: ADT: androgen deprivation therapy; ALT: alanine transaminase; ANC: absolute neutrophil count; AST: aspartate aminotransferase; BPI-SF: Brief Pain Inventory (Short Form); CES-D: Center for Epidemiological Studies-Depression; CG: control group; CI: confidence interval; CT: computed tomography; CTCAE: Common Terminology Criteria for Adverse Events; EAU: European Association of Urology; ECOG: Eastern Cooperative Oncology Group; EORTC: European Organisation for Research and Treatment of Cancer; ESTRO: European Society for Radiation Oncology; FACT-G: Functional Assessment of Cancer Therapy-General; GnRH: gonadotropin-releasing hormone; Gy: Gray; H&P: history and physical examination; HRQoL: health-related quality of life; IG: intervention group; IQR: interquartile range; LHRH: luteinizing hormone-releasing hormone; M: metastasis; MDASI: MD Anderson Symptom Inventory; MDT: metastasis-directed therapy; mpMRI: multiparametric magnetic resonance imaging; MRI: magnetic resonance imaging; N: node; NA: not applicable; NI: no information; NP: not published; OMD: oligometastatic disease; PCa: prostate cancer; PET: positron emission tomography; PFS: progression-free survival; PS: performance status; PSA: prostate-specific antigen; PSA-DT: prostate specific antigen doubling time; PSMA: prostate-specific membrane antigen; QLQ-C30: Quality of Life Questionnaire-Core 30; QLQ-PR25: Quality of Life Questionnaire-Prostate 25; QoL: quality of life; RCT: randomized controlled trial; RECIST: Response Evaluation Criteria in Solid Tumors; RT: radiotherapy; SABR: stereotactic ablative radiotherapy; SBRT: stereotactic body radiotherapy; SF-12: 12-Item Short Form Health Survey; SI: International System of Units; SIOG: International Society of Geriatric Oncology; SOC: standard of care; ULN: upper limit of normal; v: version; vs: versus; WHO: World Health Organization

^a^Testosterone in ng/mL converted to SI unit nmol/L

^b^PSA in ng/mL converted to SI unit µg/L

^c^Time from end of latest curative treatment of primary tumor to OMD

^d^Calculated from number of and time between fractions

^e^PSA progression defined as ≥25% and ≥2 µg/L increase above nadir confirmed after ≥4 weeks.^P^ Clinical progression defined as symptomatic progression (worsening symptoms or new complications^P^). Radiographic progression defined as ≥20% increase in sum diameter of soft-tissue target lesions according to RECIST 1.1 on CT, ≥1 new bone lesion(s)^P^ on bone scan or progression on MRI. If a lesion did not meet RECIST 1.1 criteria, progression was assessed by the application of RECIST 1.1 to all the lesions. Overall, any evidence of progression by size was considered progression.

^f^Local progression of soft tissue lesions defined as ≥20% and ≥5 mm increase of longest lesion diameter. Suspicious lesions on choline PET-CT were target lesions. Progression of bone lesions was defined as ≥25% increase in size of a measurable or ill-defined lesion on CT according to MD Anderson Cancer Center criteria [20].

^g^Testosterone in ng/dL converted to SI unit nmol/L

^h^Reported in ng/dL by the study authors, but assumed in ng/mL

^i^Information kindly provided by the study authors through personal correspondence

^j^Assumed reported in ng/mL by the study authors

^k^PSA progression defined as rising values with a minimum interval of 1 week and ≥2 µg/L. Radiographic progression defined as 20% increase in the sum of longest diameters of target lesions, new soft tissue or visceral lesion, and/or unequivocal progression on CT or MRI.^P^

^l^Bilirubin in mg/dL converted to SI unit µmol/L

^m^Some participants may have received conventional fractionation only, e.g., due to regional nodal disease

^n^PSA progression defined as ≥25% and ≥2 µg/L increase above nadir. Clinical progression defined as need for change of treatment (e.g., due to new symptoms) according to treating clinicians.^P^ Radiographic progression defined according to RECIST 1.1.^P^

^o^Radiographic progression defined according to RECIST 1.1 (bone scan was considered). PSA progression was considered. Clinical progression counted as event when progression could not be determined radiographically or by PSA.^P^

***References***

1. Ost P, Reynders D, Decaestecker K, Fonteyne V, Lumen N, DeBruycker A, et al. Surveillance or metastasis-directed therapy for oligometastatic prostate cancer recurrence: a prospective, randomized, multicenter phase II trial. J Clin Oncol. 2018;36:446-53.

2. Ost P, Reynders D, Decaestecker K, Fonteyne V, Lumen N, Bruycker AD, et al. Surveillance or metastasis-directed therapy for oligometastatic prostate cancer recurrence (STOMP): five-year results of a randomized phase II trial. J Clin Oncol. 2020;38:10.

3. Deek MP, Van der Eecken K, Sutera P, Deek RA, Fonteyne V, Mendes AA, et al. Long-term outcomes and genetic predictors of response to metastasis-directed therapy versus observation in oligometastatic prostate cancer: analysis of STOMP and ORIOLE trials. J Clin Oncol. 2022;40:3377-82.

4. Decaestecker K, De Meerleer G, Ameye F, Fonteyne V, Lambert B, Joniau S, et al. Surveillance or metastasis-directed therapy for oligometastatic prostate cancer recurrence (STOMP): study protocol for a randomized phase II trial. BMC Cancer. 2014;14:671.

5. Cornford P, Bellmunt J, Bolla M, Briers E, De Santis M, Gross T, et al. EAU-ESTRO-SIOG guidelines on prostate cancer. Part II: Treatment of relapsing, metastatic, and castration-resistant prostate cancer. Eur Urol. 2017;71:630-42.

6. Palma DA, Olson R, Harrow S, Gaede S, Louie AV, Haasbeek C, et al. Stereotactic ablative radiotherapy versus standard of care palliative treatment in patients with oligometastatic cancers (SABR-COMET): a randomised, phase 2, open-label trial. Lancet. 2019;393:2051-8.

7. Olson R, Senan S, Harrow S, Gaede S, Louie A, Haasbeek C, et al. Quality of life outcomes after stereotactic ablative radiation therapy (SABR) versus standard of care treatments in the oligometastatic setting: a secondary analysis of the SABR-COMET randomized trial. Int J Radiat Oncol Biol Phys. 2019;105:943‐7.

8. Palma DA, Olson R, Harrow S, Gaede S, Louie AV, Haasbeek C, et al. Stereotactic ablative radiotherapy for the comprehensive treatment of oligometastatic cancers: long-term results of the SABR-COMET phase II randomized trial. J Clin Oncol. 2020;38:2830-8.

9. ClinicalTrials.gov: Stereotactic ablative radiotherapy for comprehensive treatment of oligometastatic tumors (SABR-COMET) (NCT01446744). https://clinicaltrials.gov/ct2/show/NCT01446744 (2011). Accessed 10 Oct 2021.

10. Phillips R, Shi WY, Deek M, Radwan N, Lim SJ, Antonarakis ES, et al. Outcomes of observation vs stereotactic ablative radiation for oligometastatic prostate cancer: the ORIOLE phase 2 randomized clinical trial. JAMA Oncol. 2020;6:650-9.

11. Radwan N, Phillips R, Ross A, Rowe SP, Gorin MA, Antonarakis ES, et al. A phase II randomized trial of observation versus stereotactic ablative radiation for oligometastatic prostate cancer (ORIOLE). BMC Cancer. 2017;17:453.

12. Francolini G, Gaetano Allegra A, Detti B, Di Cataldo V, Caini S, Bruni A, et al. Stereotactic body radiation therapy and abiraterone acetate for patients affected by oligometastatic castrate-resistant prostate cancer: a randomized phase II trial (ARTO). J Clin Oncol. 2023;41:5561-8.

13. Scher HI, Morris MJ, Stadler WM, Higano C, Basch E, Fizazi K, et al. Trial design and objectives for castration-resistant prostate cancer: updated recommendations from the Prostate Cancer Clinical Trials Working Group 3. J Clin Oncol. 2016;34:1402-18.

14. Tang C, Sherry AD, Haymaker C, Bathala T, Liu S, Fellman B, et al. Addition of metastasis-directed therapy to intermittent hormone therapy for oligometastatic prostate cancer: the EXTEND phase 2 randomized clinical trial. JAMA Oncol. 2023;9:825-34.

15. Khoo V, Kirby A, Ahmed M, Dewan M, Van As N, Franks K, et al. CORE - standard of care +/- stereotactic body radiotherapy for oligometastases - primary results. Radiother Oncol. 2023;182 Suppl 1:S627.

16. The Institute of Cancer Research: CORE: a randomised trial of conventional care versus radioablation (stereotactic body radiotherapy) for extracranial oligometastases. https://www.icr.ac.uk/our-research/centres-and-collaborations/centres-at-the-icr/clinical-trials-and-statistics-unit/our-research/clinical-trials/core (2024). Accessed 25 Mar 2024.

17. Schellenberg D, Gabos Z, Duimering A, Debenham BJ, Fairchild A, Huang F, et al. Stereotactic ablative radiotherapy for oligo-progressive cancers: results of the randomized phase II STOP trial. Int J Radiat Oncol Biol Phys. 2023;117 Suppl:S58.

18. ClinicalTrials.gov: Stereotactic radiotherapy for oligo-progressive metastatic cancer: a randomized phase II trial (STOP) (NCT02756793). https://clinicaltrials.gov/study/NCT02756793 (2016). Accessed 17 Feb 2024.

19. International Clinical Trials Registry Platform: Stereotactic radiotherapy for oligo-progressive metastatic cancer (the STOP trial): a randomized phase II trial. https://trialsearch.who.int/Trial2.aspx?TrialID=NCT02756793 (2016). Accessed 17 Feb 2024.

20. Costelloe CM, Chuang HH, Madewell JE, Ueno NT. Cancer response criteria and bone metastases: RECIST 1.1, MDA and PERCIST. J Cancer. 2010;1:80-92.
